# CNS-Targeting Therapies for Lysosomal Storage Diseases: Current Advances and Challenges

**DOI:** 10.3389/fmolb.2020.559804

**Published:** 2020-11-12

**Authors:** Mariola J. Edelmann, Gustavo H. B. Maegawa

**Affiliations:** ^1^Department of Microbiology and Cell Science, The University of Florida's Institute of Food and Agricultural Sciences, University of Florida, Gainesville, FL, United States; ^2^Department of Pediatrics, College of Medicine, University of Florida, Gainesville, FL, United States

**Keywords:** lysosomes, small molecules, therapy, enzyme replacement therapy, extracellular vesicles, exosomes, liposomes, gene therapy

## Abstract

During the past decades, several therapeutic approaches have been developed and made rapidly available for many patients afflicted with lysosomal storage disorders (LSDs), inborn organelle disorders with broad clinical manifestations secondary to the progressive accumulation of undegraded macromolecules within lysosomes. These conditions are individually rare, but, collectively, their incidence ranges from 1 in 2,315 to 7,700 live-births. Most LSDs are manifested by neurological symptoms or signs, including developmental delay, seizures, acroparesthesia, motor weakness, and extrapyramidal signs. The chronic and later-onset clinical forms are at one end of the continuum spectrum and are characterized by a subtle and slow progression of neurological symptoms. Due to its inherent physiological properties, unfortunately, the blood-brain barrier (BBB) constitutes a significant obstacle for current and upcoming therapies to achieve the central nervous system (CNS) and treat neurological problems so prevalent in these conditions. To circumvent this limitation, several strategies have been developed to make the therapeutic agent achieve the CNS. This narrative will provide an overview of current therapeutic strategies under development to permeate the BBB, and address and unmet need for treatment of the progressive neurological manifestations, which are so prevalent in these inherited lysosomal disorders.

## Lysosomal Storage Diseases: Inborn Organelle Disorders Predominantly Affecting the CNS

**Figure 1:**
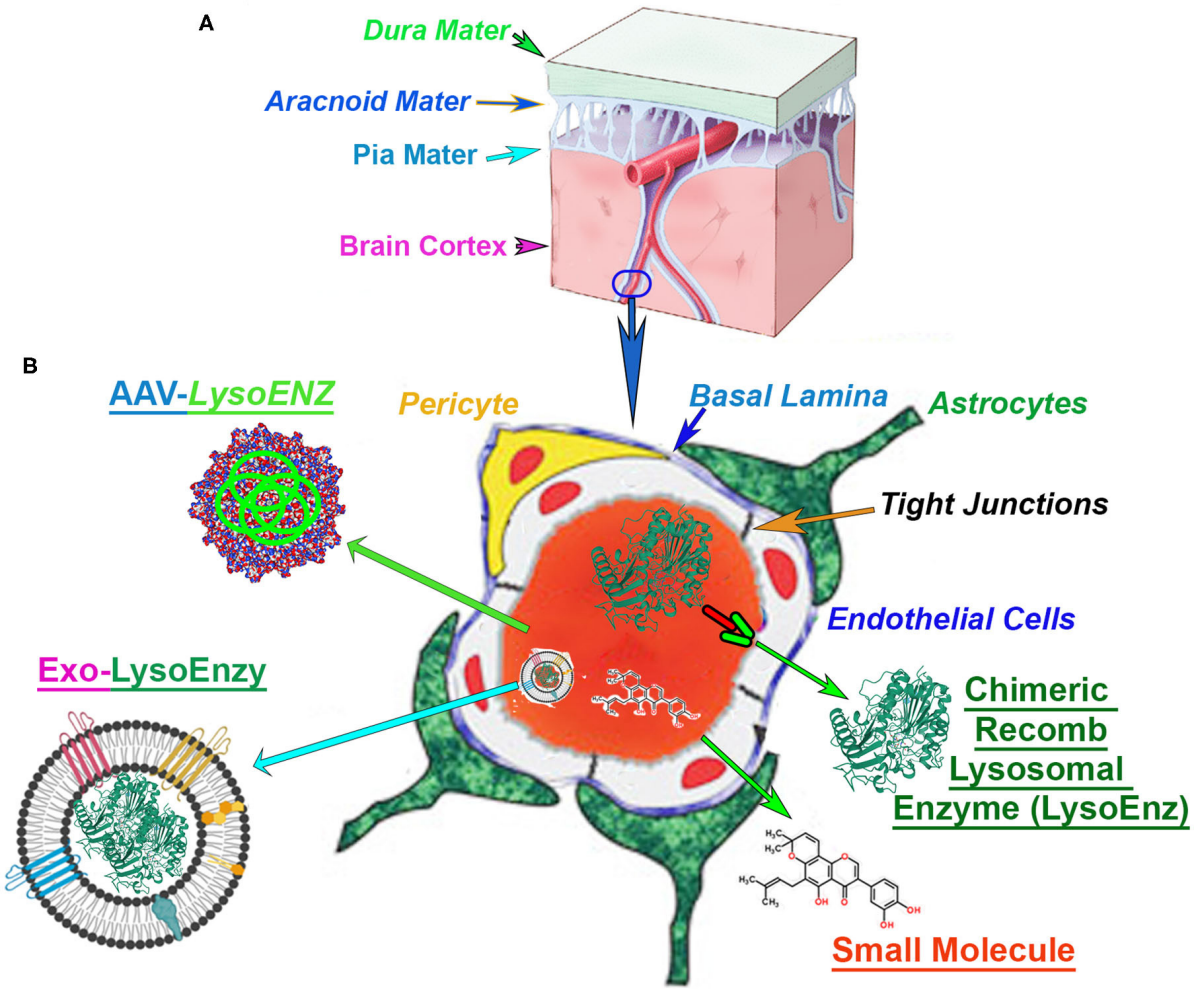
Blood-brain barrier (BBB) in the context of CNS-targetting therapeutics. **(A)** The central nervous system (CNS), and the cerebrovascular system are protected by a series of robust membranes as dura, arachnoid and pia mater. **(B)** The components of the BBB are established by the dynamic relationship among astrocytes endfeet, basal membrane, pericytes, endothelial cells and the tight junctions between the brain-endothelial cells. An illustration of the cross-section of a CNS-capillary shows the BBB components (italized), and current therapies under investigation. The modified chimeric lysosomal enzymes (LysoEnz), small molecules, gene therapy exploring specific AAV serotypes, and nanoparticle-based therapies (Exo-LysoEnzy) are designed to overcome the BBB and tackle the neuropathogenesis processes prevalent in many LSDs.

Lysosomal storage diseases (LSDs) are inborn organelle disorders characterized by multisystemic and progressive manifestations, being most of them neurological in nature (Table 1) (Patil and Maegawa, 2013; Maegawa, 2019). Intravenous enzyme replacement therapy (ERT), the mainstay treatment for several LSDs, does not address the neurological problems, as these recombinant proteins, large molecular-weight molecules, are unable to permeate through the blood-brain barrier (BBB) effectively. Several novel therapeutic agents such as intrathecal or intracerebroventricular delivery of enzymes, fusion proteins that cross the BBB, substrate reduction therapy (SRT), pharmacological chaperones (PCs), and gene therapy, are currently being developed to treat the neurological manifestations of LSDs (Figure 1).

**Table 1:** Enzyme-deficiency LSDs, classified according to primary substrate storage.

**Disease**	**Deficient Enzyme**	**Primary storage metabolite**	**Gene, locus (inheritance)**	**Neurological Symptomatology**	**Non-neurological Symptoms**
**COMMON NEUROLOGICAL LSDs**					
Gaucher disease (Types I, II, III)	Glucosylceramidase	glucosylceramide	*GBA* 1q21 (AR)	Type I–(adult) parkinsonism, peripheral neuropathy Type II–(fetus−12-months) stridor, oculomotor apraxia, dysphagia, dystonia, pyramidal tract signs, and sometimes *opisthotonus* Type III–(>1-year old) myoclonic and tonic-clonic seizures, horizontal supranuclear gaze palsy, ataxia[1-4]	Hematological–anemia, leucopenia, thrombocytopenia Visceromegaly–hepatosplenomegaly General–low energy Cardiac–pulmonary hypertension Dermatological–neonatal ichthyosis
Fabry disease	α-Galactosidase A	globotriasylceramide	*GLA* Xq22 (X-linked)	Acroparesthesia, dysesthesia, recurrent acute and chronic pain, hearing impairment, tinnitus, recurrent cerebrovascular disease	Renal–chronic renal disease Cardiac–hypertrophic cardiomyopathy arrhythmias, Ocular–corneal *verticillata* Dermatological–angiokeratomas, distal edema (hands and feet)
Pompe Disease	α1,4-glucosidase (acid maltase)	glycogen	*GAA* 17q25.3 (AR)	Lower limb-girdle and truncal muscular weakness combined with exercise intolerance migraines, dysphagia	Hypertrophic cardiomyopathy Arrhythmias aortic and cerebral vessels leading to stiffness and dilatation of ascending thoracic aorta or strokes
**MUCOPOLYSACCHARIDOSES (MPSs)**					
MPS I (Hurler, Scheie, Hurler/Scheie)	α-Iduronidase	Dermatan sulfate, heparan sulfate	*IDUA* 4p16.3 (AR)	Global developmental delay, carpal tunnel syndrome, myelopathies, spinal cord compression	Skeletal–dysostosis multiplex and multiple joint contractures Organomegaly Obstructive Sleep Apnea Corneal clouding Cardiac valvulopathies and hypertrophic cardiomyopathy
MPS II (Hunter)	Iduronate sulphatase	Dermatan sulfate,heparan sulfate	*IDS* Xq28 (X-linked)	Global developmental delay, carpal tunnel syndrome, myelopathies, spinal cord compression	Skeletal–dysostosis multiplex and multiple joint contractures Organomegaly Obstructive Sleep Apnea Corneal clouding Cardiac valvulopathies and hypertrophic cardiomyopathy
MPS III Sanfilippo syndrome	MPS-IIIA–heparan sulphamidase	Heparan sulfate	*SGSH* 17q25.3 (AR)	Global neurodevelopmental delay (1st stage), behavior problems characterized by temper tantrum, aggressive behavior, and extreme restlessness (2nd stage). Severe dementia, decline motor functions (3rd stage)	Coarse facies, obstructive air way, *dysostosis multiplex* (thoracolumbar kyphosis, abnormally shaped vertebrae and ribs, spatulate ribs, hypoplastic epiphyses, thickened diaphyses, and bullet-shaped metacarpals). Brain MRI with ventricular dilatation and enlargement of subarachnoid spaces, thin corpus callosum, enlarged perivascular spaces
	MPS-IIIB–acetyl α-glucosaminidase	Heparan sulfate	*NAGLU* 17q21.2 (AR)		
	MPS-IIIC–acetyl CoA: α-glucosaminide N-acetyltransferase	Heparan sulfate	*HGSNAT* 8p11.21 (AR)		
	MPS-IIID N-acetyl glucosamine-6-sulphatase	Heparan sulfate	*GNS* 12q14.3 (AR)		
MPS IVA (Morquio A)	Acetyl galactosamine-6-sulphatase	Keratan sulfate,chondroiotin 6-sulfate	*GALNS* 16q24.3 (AR)	Normal neurodevelopmental, carpal tunnel syndrome, myelopathies, spinal cord compression	Skeletal–dysostosis multiplex and multiple joint contractures Organomegaly Obstructive Sleep Apnea Corneal clouding Cardiac valvulopathies and hypertrophic cardiomyopathy
MPS IV B (Morquio B)	β-Galactosidase	Keratan sulfate	*GLB1* 3p22.3 (AR)	Global developmental delay, carpal tunnel syndrome, myelopathies, spinal cord compression	Skeletal–dysostosis multiplex and multiple joint contractures Organomegaly Obstructive Sleep Apnea Corneal clouding Cardiac valvulopathies and hypertrophic cardiomyopathy
MPS VI (Maroteaux-Lamy)	Acetyl galactosamine 4-sufatase (arylsulfatase B)	Dermatan sufate	*ARSB* 5q14.1 (AR)	Normal neurodevelopmental, carpal tunnel syndrome, myelopathies, spinal cord compression	Skeletal–dysostosis multiplex and multiple joint contractures Organomegaly Obstructive Sleep Apnea Corneal clouding Cardiac valvulopathies and hypertrophic cardiomyopathy
MPS VII (Sly)	β-Glucuronidase	Dermatan sulfate, heparan sufate, chondroiotin 6-sulfate	*GUSB* 7q11.21 (AR)	Global developmental delay, carpal tunnel syndrome, myelopathies, spinal cord compression	Skeletal–dysostosis multiplex and multiple joint contractures Organomegaly Obstructive Sleep Apnea Corneal clouding Cardiac valvulopathies and hypertrophic cardiomyopathy
**GLYCOSPHINGOLIPIDOSIS**					
Niemann-Pick (type A, type B)	Acid sphingomyelinase	Sphingomyelin	*SMPD1* 11p15.4 (AR)	Psychomotor development progresses no further than the 12-months level, after which neurologic deterioration is relentless	NP-A: hepatosplenomegaly with progressive hypersplenism and stable liver dysfunction, interstitial pulmonary disease, osteopenia, atherogenic lipid profile
Niemann-Pick C Disease	NPC1 and NPC2	Unesterified cholesterol and several glycosphingolipids	*NPC1 and NPC2* (AR)	Psychomotor development progresses no further than the 12-month level, after which neurologic deterioration is relentless	NP-A: hepatosplenomegaly with progressive hypersplenism and stable liver dysfunction, interstitial pulmonary disease, osteopenia, atherogenic lipid profile
Farber Disease	Acid ceramidase	Ceramide	*ASAH1* 8p22 (AR)	Severe progressive impairment of psychomotor development and neurologic deterioration with epilepsy	progressively deformed joints, subcutaneous nodules, and progressive hoarseness (laryngeal involvement) Upper airway obstruction
Gangliosidosis G_M1_(Types I, II, III)	G_M1_-β-galactosidase	G_M1_ ganglioside,Keratan sulfate,oligos, glycolipids	*PSAP* 10q22.1 (AR)	Type I: rapidly progressive with hypotonia, severe Type II: neurodegeneration extrapyramidal signs, gait disturbance	Short stature, kyphosis, and scoliosis of varying severity Cardiomyopathy
Gangliosidosis G_M2_,	β-Hexosaminidase A (Tay-Sachs) β-Hexosaminidase A + B (Sandhoff)	G_M2_ ganglioside,oligos, glycolipids	*HEXA* 15q23 (AR) *HEXB* 5q13 (AR)	Progressive weakness, loss of motor skills, decreased attentiveness, and increased startle response at 3–6 months with seizures, blindness, spasticity. In late-onset forms: progressive dystonia, spinocerebellar degeneration, motor neuron disease, and, in some individuals with adult-onset disease, a bipolar form of psychosis	Hepatosplenomegaly, coarse facial features, cardiac involvement, cherry red spot and *dysostosis multiplex*
**LEUKODYSTROPHIES**					
Krabbe	β-Galactosylceramidase (GALC)	Galactosylceramide, galactosylsphingosine (psychosine)	*GALC* 14q31.3 (AR)	Infantile onset: progressive leukodystrophy with a classical infantile-onset associate with severe neurologic impairment and deterioration and ultimately death by 2 years of ageLate onset GLD: neuropsychiatric disturbances, motor weakness, vision loss, and intellectual regression	Most symptoms are related to the neurological complications including dysphagia, recurrent pneumonias and multiple joint contractures
Metachromatic Leukodystrophy	Arylsufatase A (ASA)	Sulfatides	*ARSA* 22q13.33	Late-infantile: motor weakness, hypotonia, clumsiness, frequent falls, toe walking and slurred speech generalized or partial seizures, hearing and visual loss and peripheral neuropathy Late onset: neuropsychiatric and behavioral disturbances, motor weakness, spasticity and incontinence and peripheral neuropathy is common	Gallbladder abnormalities (polyposis, wall thickening, cholelithiasis, sludge)
Multiple Sufatase Deficiency	Multiple sulfatase	Sulfatides, glycolipids, GAGs	*SUFM1* 3p26.1 (AR)	Hypotonia, developmental regression and progressive neurodegeneration, nystagmus, dysmyelinating motor sensory neuropathy	Coarse facial features, visceromegaly, corneal clouding, upper airway obstruction, *dysostosis multiplex*
**OLYGOSACCHARIDOSES (GLYCOPROTEINOSES)**					
Aspartylglycosaminuria	Glycosylasparaginase	Aspartylglucosamine	*AGA* 4q34.3 (AR)	Speech delay, behavioral disturbances, extra-pyramidal signs with incoordination and ataxic gait, seizures	Growth spurt in infancy, gingival hypertrophy, angiokeratomas, recurrent respiratory infections are
Fucosidosis	α-Fucosidase	Glycoproteins, glycolipids, Fucoside-rich oligosaccharides	*FUCA1* 1p36.11 (AR)	Seizures, cognitive impairment, seizures, spasticity and motor weakness	Coarse facial features, short stature, *dysostosis multiplex, angiokeratoma corporis diffusum*, hepatosplenomegaly, upper airway obstruction, recurrent pneumonias
α-Mannosidosis	α-Mannosidase	Mannose-rich oligosaccharides	*MANSA* 19p13.2 (AR)	Incoordination, ataxic gait, metabolic myopathy, and incoordination. Spastic paraplegia spasticity, rigidity, and dyskinesia slight strabismus, hydrocephalus sensorineural deafness	Facial coarseness, lumbar gibbous, hepatomegaly, and *dysostosis multiplex*
Schindler disease	*N*-acetylgalactosaminidase	Sialylated asialoglycopeptides, glycolipids	*NAGA* 22q13.2 (AR)	Neuroaxonal dystrophy, moderate psychomotor retardation, autistic features	Profuse *angiokeratoma corporis diffusum*, hepatomegaly and cardiomyopathy
Sialidosis	Neuraminidase	Oligos, glycopeptides	*NEU1* 6p21.33 (AR)	Developmental delay, myoclonic epilepsy, visual impairment and ataxia, generalized tonic-clonic or myoclonic seizures, progressive visual impairment along with night blindness, nystagmus	*hydrops fetalis*, retinal cherry-red spot, coarse facial features, hepatomegaly, *dysostosis multiplex*, corneal opacities
Mucolipidosis IIα/β, IIIα/β	GlcNAc-1-P transferase	Oligos, GAGs, lipids	*GNPTAB* 12q23.2 (AR)	Developmental delay in motor milestones with preservation of receptive and expressive speech, stuttering, seizures, motor weakness	*dysostosis multiplex*, corneal cloudiness, cardiac valvulopathies (aortic and mitral valve), obstructive sleep apnea
Mucolipidosis IV	Mucolipin	Sulphatides, glycolipids, GAGs	*TRMPL1* 19p13.2 (AR)	Neuropsychomotor delay and subsequent visual impairment, speech delay (receptive language is better than expressive language) progression to severe dysarthria or anarthria, slow chewing, slow eating and swallowing, and spastic diplegia or quadriplegia. Hypotonia, hyperreflexia and spasticity	corneal clouding, retinal degeneration
Wolman/CESD	Acid lipase	Cholesterol esters	*LIPA* 10q23.31 (AR)	Wolman disease form:Cognitive impairmenthypotoniabilateral ptosis and external ophthalmoplegia	Hepatosplenomegaly Jaundice, steatosis, fibrosis, cirrhosis and and/or liver failure) atherosclerosis (coronary artery disease, stroke) hypersplenism (i.e., anemia and/or thrombocytopenia) malabsorption
Galactosialidosis	Protective protein cathepsin A (PPCA)	Sialyloligosaccharides	*CTSA* 20q13.12 (AR)	Hypotonia, spasticity and seizures, cognitive decline	*hydrops fetalis*, angiokeratomas, hepatosplenomegaly, coarse facial features, cardiomyopathy, ‘cherry red spot' and *dysostosis multiplex*
Danon Disease	Lysosome-associated membrane protein 2 (LAMP-2)	cytoplasmatic debris and glycogen	*LAMP2A* Xq24	Motor weakness and muscle atrophy are predominantly noted in proximal musculature as shoulder girdle muscles, however, some patients may present distal muscles. Elevated serum levels of creatine kinase (CK) are elevated as well as in 50–60% of female patients	Cardiomyopathy

*AR, autosomal recessive*.

The unmet need for more efficacious drug delivery to the central nervous system (CNS) is currently the primary problem and often attribute to the properties of the BBB, considered the “problem behind the problem” when developing therapies for neurological conditions (Pardridge, 2005). The BBB is a term used to describe the unique properties of the microvasculature of the CNS, constituted by continuous non-fenestrated vessels, containing a series of additional “gatekeepers” tightly regulating the movement of molecules, ions, and cells between the blood and its neural cells (Figure 1) (Daneman and Prat, 2015). Neurodegenerative disorders such as Alzheimer's, Parkinson's, and multiple sclerosis, among others, constitute ~17% of total deaths globally (GBD 2016 Neurology Collaborators Group, 2017). The majority of the promising drugs with the potential to treat CNS disorders, including mostly small molecules, fall in the late pre-preclinical phase due to their failure to achieve therapeutic levels in the brain. In fact, among the current small molecule drugs approved, only 2% or less can produce significant trough levels in CNS and used therapeutically to treat different neurobehavioral manifestations. Those are mostly antipsychotics, mood stabilizers, anti-depressants, and anti-epileptics (Ghose et al., 2012). Many orphan disorders also manifest with significant progressive neurodegeneration and have served as models for the development of novel strategies to address the BBB drug delivery challenge (Begley et al., 2008). The efficacy of therapeutic options depends on the bioactivity of the therapeutic agent, but equally important is its ability to reach critical targets within tissues and cells affected by the disease processes. Intravenous administration is the simplest and least invasive method for systemic delivery of drugs by infusing molecules directly into the circulatory system. The blood supplies all cells and tissues with vital elements and nutrients. However, the delivery of macromolecules such as proteins from the circulation into adjacent cell layers is limited by a variety of biological barriers and selectivity of cell uptake mechanisms. The macromolecular transport across plasma and intracellular membranes are regulated by integral transmembrane channels or carriers. For any particular drug, the specific components required for uptake might be limited or lacking, especially in specialized tissues such as the endothelium of the CNS. Exogenous molecules interface with at least three distinct barriers that modulate transport into the brain: BBB, the blood-cerebrospinal fluid (CSF) barrier, and the arachnoid barrier (Begley et al., 2008; Abbott et al., 2010). The BBB is formed by vascular endothelial cells and other neural cells making a specialized network of capillaries with an extensive interface for blood-brain exchange with a total surface area of 12–18 m^2^ in humans (Pardridge, 2001, 2007a; Begley et al., 2008). Molecules in the blood gain access to the brain by exploiting selective cell surface transporters or by free diffusion if the molecule is lipophilic and has a molecular weight <400 Da (Pardridge, 2006; Sweeney et al., 2018). In response to the challenges imposed by BBB to treat neurological manifestations which are prevalent in several LSDs, a number of strategies to deliver small molecules and biological agents to the CNS include neurosurgical-based interventions, chemical-based strategies (e.g., promoting lipid solubility of the molecules) (Corraliza-Gomez et al., 2019); and biology-based strategies (e.g., vesicular mechanism regulated by endogenous BBB transporters) (Sweeney et al., 2018).

## Perspectives on the CNS-Targeting Therapies and Their Limitations

Unfortunately, for the vast majority of the neurological forms of LSDs, the core of patient management is based on multidisciplinary supportive care, which includes addressing the disease-related neurological complications with neurosurgical procedures. The classical example is the decompression of the cervical spine and the placement of ventricular-peritoneal shunt for hydrocephalus (Muenzer et al., 2009b). In addition, the use of anticonvulsants for seizures and assistance for patients with learning disabilities, orthopedic interventions to alleviate spinal deformities, joint limitations, and retractions; nutritional support; cardiac surgery when involvement of cardiac valves or coronaries are part of the complex care required in management of patients diagnosed with LSDs.

Over recent years, intensive and continual efforts have been made to develop therapies to tackle the neuro-pathogenesis of these inborn organelle disorders (Alderson et al., 2019). The approaches developed to treat LSDs are based on different strategies, each directed to manipulate a specific event in the downstream pathogenic cascades. The majority of these approaches target the deficiency of lysosomal enzyme/protein and/or the accumulated substrate. The former can be achieved by administering the recombinant human lysosomal enzyme, or by taking advantage of the ability of cells to uptake the agent through the mannose 6-phosphate receptor (Ghosh et al., 2003), delivering it to the lysosomal compartment. This approach is the enzyme replacement therapy (ERT) and is currently the core therapeutic modality used in the majority of LSDs, but limited to treat non-neurological, or somatic, manifestations of these conditions (Platt, 2018).

More recently, to address the poor biodistribution of the agent to certain organs, including CNS studies on modifying the ERT agent have emerged (Condori et al., 2016) and skeletal muscle (Koeberl et al., 2011; Peng et al., 2017; Han et al., 2019). To investigate the potential of large-molecular weight compounds to treat neurological disorders, novel approaches are required to surmount the BBB. Based on the ability of the adsorptive-mediated endocytosis of plant RTB-lectin, plant-based bioproduction of a fusion enzyme of ERT agent and lectin were accomplished, preserving the lysosomal enzymatic activity (Acosta and Cramer, 2020). Two murine LSD models, *IDUA*^−/−^ (MPS I), GM1 gangliosidosis mice, treated with the ERT agent-RTB-lectin showed significant normalization of substrate levels in CNS, and correction of learning and memory deficits of the mouse model (Condori et al., 2016; Ou et al., 2018). Other studies have shown that the cellular uptake of α-iduronidase (IDUA), the enzyme deficient in MPS I, is enhanced by a fusion of IgG-IDUA fusion protein, where the IgG domain is a genetically engineered monoclonal antibody (MAb) against the human insulin receptor (HIR) (Boado et al., 2008, 2013). Based on these studies on murine *IDUA*^−/−^ (MPS I), a phase 1/2 trial was performed in which patients affected by MPS I received the HIRMAb–IDUA fusion protein by intravenous infusion for 52-weeks DQ, and the cortical gray matter volume of the brain were stabilized by valanafusp alpha treatment. Somatic manifestations were stabilized, or improved, based on urinary glycosaminoglycan levels, hepatic and spleen volumes, and shoulder range of motion (Giugliani et al., 2018). The adverse events were dominated by transient hypoglycemia in 6.4% of subjects enrolled (Giugliani et al., 2018). Further studies will be necessary in earlier symptomatic or presymptomatic patients with the neuronopathic predictive genotype of MPS-I, as well as, studies in other neurological lysosomal storage disorders are needed. In preclinical studies, two fusion candidates, IDUAe1 and IDUAe2, were identified and shown to be desirable receptor-mediated binding, endocytosis, and transendothelial transport as well as appropriate lysosomal enzyme trafficking and biological function (Wang et al., 2013). The increased peripheral *IDUAe1* or *IDUAe2* through the hepatic expression resulted in BBB capillary-depleted endothelial cells and protein delivery into non-endothelium perivascular cells, neurons, and astrocytes in the murine MPS-I model, achieving levels of 2–3% of normal brain IDUA activities (Wang et al., 2013). Using the mucopolysaccharidosis type IIIA (MPS-IIIA) murine model, a neurological MPS caused by the *N*-sulfoglucosamine sulfohydrolase deficiency (Table 1), studies on a chimeric sulphamidase, containing a signal peptide from Apolipoprotein B (ApoB-BD), showed a highly secreted iduronate-2-sulphatase (IDS) and efficient BBB transcytosis and restoration of sulphamidase activity in the brain of treated mice (Sorrentino et al., 2013).

Alternatively, the recombinant enzyme can be secreted by donor stem-cell-derived cells or genetically manipulated cells from the patient. This strategy is the hematopoietic stem-cell therapy (HSCT) and is efficacious for a few neurological LSDs including the neuronopathic forms of mucopolysaccharidosis type I (MPS-I) (Wiseman et al., 2013). In a recent study, a brain-targeted hematopoietic stem cell gene therapy approach using lentiviral IDS fused to ApoEII (IDS.ApoEII) compared to a lentivirus expressing wild-type (normal) IDS to standard bone marrow transplant. In mucopolysaccharidosis II mice, only IDS.ApoEII mediated complete normalization of brain pathology and behavior, providing significantly enhanced correction compared to IDS. Gleitz et al. (2018) In addition, the corrected macrophages traffic to the brain, secreting IDS/IDS.ApoEII enzyme for cross-correction. The IDS.ApoEII enzyme showed to be more active in plasma and was taken up by endothelial cells, and transcytosed across the BBB at a significantly higher rate than the native IDS via both heparan sulfate/ApoE-dependent receptors and mannose-6-phosphate receptors (Gleitz et al., 2018). Recently, lentiviral vector-mediated expression of the chimeric GALC enzymes was shown to be safe and leads to supranormal enzymatic activity in both neural and hematopoietic cells. The IDSsp.GALC showed enhanced expression and secretion in comparison to the unmodified GALC. The chimeric GALC enzymes produced by LV-transduced cells reduce intracellular galactosylceramide (GalCer) storage and effectively cross-correct GLD murine neurons and glial cells. These findings suggest that ERT of chimeric GALC can be used by the affected CNS cells and tissues, supporting the development of novel and more effective GT approaches for GLD (Ricca et al., 2020).

The gene encoding the deficient lysosomal enzyme can be delivered to the cells through the use of AAV systems. Subsequently, patients cells can synthesize the normal lysosomal enzymatic cells (Biffi, 2017a,b). This approach is known as *ex-vivo* gene therapy. Another approach is to restore the equilibrium between the synthesis of the accumulated, or “stored,” substrates, and their limited degradation due to the lysosomal enzymatic deficiency (Platt and Jeyakumar, 2008; Marshall et al., 2016). This strategy is called substrate reduction therapy (SRT), which is achieved by modulating the primary substrate biosynthesis with small molecule inhibitors of critical limiting steps. The “reduction of the accumulated substrate” can also be achieved by promoting the clearance of primary and secondary accumulated products from the affected cells (Gatto et al., 2017; Torra et al., 2018).

In this review, we focus on current treatment strategies for LSDs targeting the CNS, including those that hold promise for future advancements in the treatment of the progressive neurological manifestations of these devastating disorders.

## Therapeutic Strategies Potentially Targeting CNS Manifestations of LSDs

### Small Molecule Therapies

Small molecules are most frequently used experimentally to modulate the function of a protein target and assess the consequences. The small molecules are low-molecular-weight (<900 Da) synthetic organic compounds that have antagonistic or agonistic effects on intra and extracellular targets. By being small molecules, these therapeutic agents are more likely to cross the BBB and achieve the CNS, and ultimately the neural cells with endolysosomal network dysfunction due to the lysosomal enzyme/protein deficiency. Examples of small molecules that have been used to treat LSDs are pharmacological chaperones (PCs), proteostasis regulators, substrate reduction therapy (SRT) agents, and drugs that increase gene expression by suppressing translation termination, also known as premature termination codon (PTC) suppressing agents. Here, we describe the current classes of small molecules that have shown evidence of BBB penetration and potentially treating some neurological manifestations of some LSDs (Table 2).

**Table 2:** Overview of approved, orphan drug designations, off-labeled therapies for treating lysosomal storage disorders, and examples of some products under development with an orphan drug designation.

**LSDs**	**Therapeutic agent**	**Type/Current status**	**Evidence of CNS penetration**
Gaucher disease (GD)	Imiglucerase (Cerezyme)	ERT-IV (produced in CHO cells)/approved^*^	None
	Velaglucerase (VPRIV)	ERT-IV (produced in human cells)/approved^*^	None
	Taliglucerase (Elelyso)	ERT-IV^(^produced in plant cells)/approved^**^	None
	miglustat (Zavesca)	SRT/approved^*^	None
	eliglustat (Cerdelga)	SRT/approved	None
	ambroxol	PC/off-labeled use neuronopathic forms of GD	Myoclonic epilepsy and cognition improvement; decreased CSF glucoSPG (Narita et al., 2016; Pawlinski et al., 2016; Charkhand et al., 2019; Kim et al., 2020)
Fabry Disease	agalsidase beta (Fabrazyme)	ERT-IV (produced in CHO cells)/approved^*^	None
	agalsidase alfa (Replagal)	ERT-IV (produced in human cells)/approved^**^	None
	migalastat (Galafold)	PC/approved^*^	None
MPS-I	Laronidase (Aldurazyme)	ERT-IV(produced in CHO cells)/approved	None
	HSCT	Cell therapy/approved	Efficacious on neuronopathic MPS-I (Prasad and Kurtzberg, 2010a,b; De Ru et al., 2011)
	Fusion-ERT	HIRMAb-IDUA-IV#/ODD	Preliminary evidence in small and short clinical studies (Giugliani et al., 2018)/ODD
MPS-II	Idursulfase (Elaprase)	ERT-IV (produced in human cells)/approved^*^	None
	Idursulfase (Elaprase)	ERT-IT (produced in human cells)/ODD	Mild effects/ODD (Muenzer et al., 2016)
	Idursulfase (Elaprase)	ERT/ODD	Mild improvement spinal cord compressions/ODD
MPS-IIIA	Sulfamidase	ERT-IT/ODD	Decline in HS in CSF/ no change in neuro endpoints/ODD
	LYS-SAF302 (Lysogene/Sarepta Therap.)	IV-GT (systemic) LYS-SAF302	No results from clinical trials NCT03612869
	scAAV9.U1a.hSGSH (Abeona Therap)	IV-GT (systemic) scAAV9.U1a.hSGSH	No results from clinical trials NCT02716246
	*Ex vivo* HSCT	CD34+-Lenti-transduced (*SGSH*)	No results from clinical trials NCT04201405
MPS-IIIB	SBC-103(rhNAGLU) (Alexion Pharm.)	ERT-IV	Negative results (Whitley et al., 2019)
	NAGLU–IGF2 fusion protein	NAGLU–IGF2 fusion protein -IV#/ODD	No results from clinical trials (Prill et al., 2019)
	rAAV9.CMV.hNAGLU	IV-GT (systemic) -rAAV9.CMV.hNAGLU/ODD	No results from clinical trials NCT03315182
	rAAV2/5-hNAGLU (UniQure Biopharma B.V.)	IT-GT (local) rAAV2/5-hNAGLU	No results from clinical trials NCT03300453
MPS-IVA Morquio Synd.	elosulfase (Vimizim),	ERT-IV (produced in CHO cells)/approved^*^	None
MPS-VI Maroteaux-Lamy	galsulfase (Naglazyme)	ERT-IV (produced in CHO cells)/approved^*^	None
MPS-VII Sly syndrome	vestronidase alfa (Mepsevii)	ERT-IV (produced in CHO cells)/approved^*^	None
Pompe Disease GSD-II	alglucosidase alfa (Lumizyme)	ERT-IV (produced in CHO cells)/approved^*^	None
	alglucosidase alfa (Myozyme)	ERT-IV (produced in CHO cells)/approved^**^	None
	avalglucosidase alfa, (neo-GAA; ATB200; Amicus)+ AT2221 (miglustat)	ERT-IV (rhGAA-ATB200)/PC (AT2221)/ODD	None (Xu et al., 2019)
Metachromatic Leukodystrophy	*Ex vivo* HSCT	GT-Lenti-transduced (*ARSA*)/ODD	Stabilization of neurocognition and white-matter signal in brain MRI studies (Sessa et al., 2016)
Globoid-cell leukodystrophy (GLD), Krabbe disease	HSCT	Cell therapy/approved	HSCT at <30 days of age, improvements in mobility, speech, oropharyngeal function (Allewelt et al., 2018)
Lysosomal acid lipase deficiency (Wolman disease/cholesteryl ester storage disease)	Sebelipase (Kanuma)	ERT-IV (produced in egg white–genetically modified chicken)/Approved^*^	None
Neuronal ceroid lipofuscinosis type 2	Cerliponase (Brineura)	ERT-IT (produced in recombinant CHO cells)/Approved^*^	Reduce progression of neuro-cognitive decline (Markham, 2017; Schulz et al., 2018)
Niemann–Pick disease type B (Acid sphingomyelinase deficiency)	olipudase alfa	ERT-IV (produced in CHO cells)	Not yet observed (Wasserstein et al., 2018)
Niemann–Pick disease type C	miglustat (Zavesca)	SRT (oral)	HSEM velocity; improvement in swallowing capacity, auditory acuity, and a slower deterioration
	Vorinostat	HDAC inhibitor/off-labeled use neuronopathic forms of GD	None NCT02124083
	2-Hydroxypropyl-β-Cyclodextrin (VTS-270)	IT-VTS-270	Stable but slower-than-average Cognitive Scales (Farmer et al., 2019)
α-Mannosidosis	velmanase alfa/(Lamzede)	ERT-IV (produced in CHO cells/approved)	None (Borgwardt et al., 2018)

* *Approved in Europe, USA, and other countries*;

** *Approved in USA, Brazil and Canada*;

^***^Approved in Europe. CHO, Chinese Hamster Ovary, CHO cells; enzyme replacement therapy; ERT, enzyme replacement therapy; glucoSPG, glucosylsphingosine; GSD, glycogen storage disease; GT, gene therapy; HIRMAb, human insulin receptor monoclonal antibody; HSEM, horizontal saccadic eye movement; HSCs, hematopoietic stem cells; HSCT, hematopoietic stem cell therapy; HSP, heat shock protein; IV, intravenous (systemic); IT, intra-thecal (local); MPS, mucopolysaccharidosis; NAGLU, alpha-N-acetylglucosaminidase; ODD, orphan drug designation; SRT, substrate reduction therapy; PC, pharmacological chaperones.

#### Pharmacological Chaperones (PC)

A number of missense pathogenic gene variants encode mutant misfolded proteins, resulting in interactions with elements of the endoplasmic reticulum (ER)-associated degradation (ERAD) pathway (Bhattacharya and Qi, 2019), and subsequent premature degradation by the endoplasmic reticulum quality control (ERQC) machinery (Maegawa et al., 2007; Balch et al., 2008; Mu et al., 2008b). Pharmacological chaperones (PCs) interact earlier with the target misfolded mutant protein in the endoplasmic reticulum, in the case of lysosomal enzymes, assisting its folding, attenuating its high-free energy (ΔG_0_), toward a lower ΔG_0_” state (Powers and Balch, 2013), passing the ERQC, and evading the ERAD (Wiseman et al., 2007). Subsequently, the properly folded mutant protein reaches the cis-Golgi network and, where specific biochemical modifications allow the interactions with mannose-6 phosphate receptors and, eventually, landing in the lysosomal compartment (Sawkar et al., 2006). Once at the lysosomes, due to its acidic environment (pH 4–5) and abundance of the undegraded and accumulated substrate, optimal PCs lose their affinity to the target mutant, leaving their “site-of-binding,” often the active-site, free for intercations with the natural enzymatic substrate (Tropak and Mahuran, 2007). Several compounds have been tested as potential PCs for the treatment of neurological symptoms of lysosomal disorders. These compounds include ambroxol (Maegawa et al., 2009b; Klionsky et al., 2016), and most recently progranulin (Jian et al., 2016) and NCGC607 for the treatment of Gaucher disease (Aflaki et al., 2016); pyrimethamine for late-onset Tay- Sachs disease (Maegawa et al., 2007; Clarke et al., 2011; Osher et al., 2011); *N*-octyl-epi-β-valienamine (Aflaki et al., 2016) and, recently, derivatives of 4-epi isofagomine for β-galactosidase deficiency, betaine for aspartylglucosaminuria (Lebl et al., 2017) and the current PC molecule approved for Fabry disease, migalastat (Germain et al., 2016; Hughes et al., 2017). Protein homeostasis, or proteostasis, consists of a network of interacting activities, or “interactome,” in charge of maintaining the “health” of the proteome and the organism (Balch et al., 2008; Song et al., 2013; Wang and Segatori, 2013). This network comprises several ER-resident chaperones and degradation elements as well as stress-responsive signaling pathways that detect the misfolding and/or aggregation of proteins in specific subcellular compartments using stress sensors, which respond by generating an active transcription factor (Roth and Balch, 2011; Klionsky et al., 2016; Kelly, 2020).

#### Proteostasis Regulators

Proteostasis regulators are defined as small molecules able to increase the residual function of lysosomal mutant enzymes by manipulating components of several folding and protein degradation signaling pathways (Kelly, 2020). By affecting and interacting with components of the interactome network, the proteostasis regulators have a broader and more pervasive outcome effect on multiple misfolded mutant proteins, which implies in a broader clinical application for several “misfolding protein disorders.” (Mu et al., 2008a; Kelly, 2020). One of the examples of proteostasis regulators is bortezomib, an approved-proteasome inhibitor approved for myeloma, which showed to decrease the levels of intracellular accumulated cholesterol in cultured cells with NPC1 deficiency from individuals with the Niemann-Pick disease type C (Macias-Vidal et al., 2014). In addition, the same proteostasis regulator showed to restore glucocerebrosidase (GCase) activity from Gaucher disease by inhibiting histone deacetylases and preventing mutant GCase degradation (Fan et al., 2011). Other histone deacetylase inhibitors, vorinostat, and panobinostat showed to decrease the cholesterol typically accumulated in human Niemann-Pick C1 fibroblasts (Pipalia et al., 2017). Also, the manipulation of ER-calcium concentrations in the ER has been investigated as a potential therapeutic approach with promising results in Gaucher disease, Niemann-Pick disease type C, and mucolipidosis type IV (Matalonga et al., 2017). However, the long-term effects of the regulation of the proteostasis cannot be predicted. The link between autophagy impairment and lysosomal disorders has led to novel approaches to modulate cellular clearance, which has been investigated in preclinical studies (Palmieri et al., 2017), but the translation to clinical trials is still premature.

#### Substrate Reduction Therapy

The substrate reduction therapy (SRT) agent consists of a small molecule that partially inhibits the biosynthesis of the natural substrate accumulated secondary to the enzymatic deficiency. It is an attractive approach to mitigate the load of the primary and eventually secondary substrate of the deficient lysosomal enzyme (Platt et al., 1994a,b). The first substrate reduction therapy agent developed for a LSD was miglustat, an imino-sugar deoxynojirimycin, and its alkylated derivatives, known inhibitors of the *N*-linked oligosaccharide processing enzymes (Platt et al., 1994b). In preclinical studies, the imino sugar *N*-butyldeoxynojirimycin showed also to interact with glucosyltransferases, especially inhibiting ceramide-specific glucosyltransferase that catalyzes the first step in glycosphingolipid biosynthesis (Platt et al., 1997). Miglustat was later approved for the treatment of Gaucher disease type I (Cox et al., 2000), and Niemann-Pick C disease (Patterson et al., 2007). Despite the reduction of the increasing levels of the glycosphingolipids in the CNS of murine models of gangliosidoses, in early human clinical trials in GM2 and GM1 gangliosidoses, the small molecule failed to achieve sufficient CNS levels to result in measurable clinical benefits (Maegawa et al., 2009a,c; Shapiro et al., 2009).

Studies using miglustat in combination with ERT for neuropathic Gaucher disease (types II and III) showed no significant alterations of clinical endpoints (Schiffmann et al., 2008). Despite the anecdotal reports on neurological improvement in some cases (Capablo et al., 2007), no SRT agents, a standard of care has been used with ERT. In this setting, and given the significant side effects of miglustat (Pastores et al., 2007; Giraldo et al., 2009), another ceramide analog, eliglustat, was developed by Dr. Shayman's group (Mceachern et al., 2007; Shayman, 2010, 2013), and later approved for the treatment of Gaucher disease type I (Cox et al., 2015; Mistry et al., 2015). However, eliglustat fails to permeate the BBB, limiting the therapeutic use to non-neuropathic symptoms of Gaucher disease. Small molecules that function as cholesterol-lowering agents have been identified for Niemann-Pick disease type C disease (Camargo et al., 2001;Davidson et al., 2009).

The intrathecal 2-hydroxypropyl-β-cyclodextrin has been shown to have clinical benefits or stabilization of disease progression in most of the 14 patients reported (Ory et al., 2017; Farmer et al., 2019). Further clinical trials with a larger number of patients with NPC studies are either being analyzed (NCT01747135) or ongoing (NCT03887533, NCT03893071) using the intrathecal and intravenous route, respectively. In terms of the mucopolysaccharidosis (MPS) I, II, and III (all subtypes), and their neurological manifestations, the genistein, an isoflavone, and the protein tyrosine kinase and epidermal growth factor inhibitor resulted in the reduction of glycosaminoglycan (GAG) accumulation (Malinowska et al., 2009). The early phase clinical studies showed a reduction of urinary excretion of GAGs and plasma heparan sulfate concentration in patients with MPS III (De Ruijter et al., 2012). However, the absolute reduction of these metabolites was small after a 12-months treatment period coinciding within the range as observed in untreated patients. Also, genistein showed no efficacy in the clinical endpoints when examining in 30 affected MPS-III enrolled in the study (De Ruijter et al., 2012). Evidence of another potential mechanism of action of the 2-hydroxypropyl-β-cyclodextrin has been described against cytotoxic psychosine, which is a sphingolipid found at high and cytotoxic levels in Krabbe disease due to the GALC deficiency in the CNS (Katabuchi et al., 2018).

#### Small Molecules Targeting Nonsense Mutations in LSDs

By definition, nonsense pathogenic genetic variants result in the premature termination codon (PTC) and subsequent translation of a truncated protein that is typically degraded by nonsense-mediated decay. An extensive meta-analysis on the Human Gene Mutation Database has revealed that approximately 11% of all described pathogenic variants resulting in human inherited disorders are caused by nonsense mutations (Mort et al., 2008; Bidou et al., 2012). Nonsense pathogenic variants are identified in several LSDs that may potentially benefit from small molecules that function as PTC suppression-stimulating agents by promoting “read-through' the premature termination or stop codon. In LSDs, chloramphenicol showed to increase the *IDUA* gene expression and, subsequent, α-L iduronidase (IDUA) activity in cell lines derived from patients with MPS-I (Mayer et al., 2013). Also, B84 suppressed the *IDUA*-W392X nonsense mutation much more efficiently than any of the other compounds tested. NB84 treatment restored enough functional α-L-iduronidase activity to partially reverse abnormal GAG accumulation and lysosomal abundance in mouse embryonic fibroblasts derived from the Idua-W392X mouse (Wang et al., 2012). In cultured oligodendrocytes from *Twitcher* mice (*galc*^*twi*/*twi*^), naturally occurring murine model for Krabbe disease, and patient fibroblasts with nonsense mutations in *GALC* gene, the nonsense-mediated mRNA decay (NMD) inhibitor 1 (NMDI1) (Durand et al., 2007) increased the levels of mRNA and rescued galactocerebrosidase (GALC) activity in a dose-dependent manner, and improve the morphology of the differentiated oligodendrocytes (Luddi et al., 2016). In infantile neuronal ceroid lipofuscinoses, using the novel Cln1(R151X) mouse model, mice receiving PTC124 (ataluren) showed enhancements of the palmitoyl-protein thioesterase 1 activity were noted, but limited to the liver and muscle tissues, alluding the challenges of biodistribution of this specific class of small molecules (Thada et al., 2016).

### Cell Therapy

The basis and rationale of using hemopoietic stem cell transplantation (HSCT) to treat LSDs originated from elegant and pioneering experiments of Neufeld and cols. who demonstrated in cultured cells of patients with different mucopolysaccharidoses, cross-correction with reductions of the accumulated GAGs were observed (Fratantoni et al., 1968; Neufeld, 1989). The HSCT potential resides in the permanent biological source (donor cells) of steady functional lysosomal enzymes secretion and their internalization through the mannose-6 phosphate receptor system by surrounding “recipient” affected cells (Platt et al., 2018). The mannose 6-phosphate receptor allows the exogenous lysosomal enzyme to directly targeting the dysfunctional and enlarged lysosomes. The hematopoietic stem cells (HSCs) can also cross the BBB and differentiate into microglial cells, and populate the CNS, becoming active sources of lysosomal enzymes (Tan et al., 2019). Numerous factors may impact the efficacy of the HSCT in LSDs and other inherited metabolic disorders. These factors can be clinical and HSCT-related, including drug regimens of conditioning pre-HSCT, presence and degree of graft-vs. host disease, time of neutropenic recovery, and, ultimately, clinical status and, especially, the disease stage of the affected patient undergoing the procedure (Biffi, 2017b). From a biological standpoint, several factors can affect the level of “cross-correction” of the deficient lysosomal enzyme, including the number of donor HSCs administered, level of differentiation once in CNS, the degree of secretion of the lysosomal enzyme by the donor HSCs, and also the properties of the lysosomal enzyme to be uptaken by the recipient neural cells. The sum of these factors will determine the outcome, which is the arrest and/or improvement of neurological manifestations of LSDs. In addition, in the setting of a generalized lysosomal dysfunction, the extracellular microenvironment is altered by the presence of a number of cytokines and inflammatory response cells, which also contribute substantially to the pathogenesis of the neurodegeneration observed in LSDs (Walkley, 2009; Platt et al., 2018). If successful, with full engraftment, the HSCT can single-intervention, offering a lifelong source of secretion of “deficient' enzyme” for the recipient patient. However, the physiological demands of lysosomal levels may change during the natural history of the LSDs. The donor HSCs are allogenic donors either from family-related members, usually siblings. Given the setting of early diagnosis and also with the newborn screen for MPS-I and Krabbe disease, the Umbilical cord blood (UCB) as an alternative stem cell source has been used in HSCT (Lund, 2018). The UCB is a safe and effective source of HSCs.

In terms of limitations, only in MPS-I (iduronate deficiency), the HSCT procedure has shown to be efficacious to prevent and attenuate the neurological manifestations of the neuronopathic forms (Ballen et al., 2013). The numbers of umbilical HSCs are usually lower than those from bone marrow sources. In pediatric patients, the UCB source can provide desired doses of HSCs number/kilogram, whereas, adults undergoing HCT may suffer from inadequate cell numbers (Ballen et al., 2013). As in an adult setting, the UBC as a source of HSCs becomes limited, several approaches have been proposed, including combined infusion of 2 UCB units (Barker et al., 2001), increasing the homing efficiency of UCB cells (Cutler et al., 2013), and expanding the UCB *in vitro* (Klionsky et al., 2016). Recently, small molecule, carlecortemcel-L, a copper chelator known for its ability to expand UCB HSCs *in vitro* (Peled et al., 2004), allowed a selected CD133+ cell expansion post-selection from UCB units, followed by a 21-days expansion with cytokines and carlecortemcel-L *in vitro* (Stiff et al., 2018).

In sum, the significant advantages of the HSCT are dependent upon a number of factors, and a higher concentration of enzymes delivered to the CNS will result in better clinical outcomes. Therefore, it is essential that HSCs source, either the UCT- or BM-derived, to be screened for the carrier status of the specific lysosomal enzyme deficiency diagnosed in the recipient patient.

Since 1980, several patients with LSDs have been treated and a wide spectrum of outcomes reported. At present, HSCT is highly recommended for patients with MPS-I if they are younger than 24 months, as per the European Union (Clarke et al., 2017), and 36-months, as per US guidelines (De Ru et al., 2011). The MPS-I is the only LSD in which full prevention of the neuropathogenic processes is observed if the HSCT is done before the neurocognitive impairment arises (Aldenhoven et al., 2015). In addition, HSCT has been beneficial for patients with metachromatic leukodystrophy (MLD) and Krabbe disease if the transplantation is performed at the presymptomatic stage (Escolar et al., 2005; Mcgraw et al., 2005; Cartier and Aubourg, 2008; Duffner et al., 2009; Krageloh-Mann et al., 2013; Tejera et al., 2013; Groeschel et al., 2016; Van Rappard et al., 2016; Allewelt et al., 2018; Kwon et al., 2018). However, cognitive decline and neurodegenerative disease are observed in a number with early-onset Tay-Sachs disease and Sandhoff disease (Boelens et al., 2014), MPS-III and MPS-II (Mckinnis et al., 1996; Guffon et al., 2009; Muenzer et al., 2009a; Scarpa et al., 2011), who underwent HSCT. Recently, in preclinical studies, encouraging results of human neural cells in several LSD murine models, including Niemann-Pick A disease (sphingomyelinase deficiency) (Shihabuddin et al., 2004), infantile neuronal ceroid lipofuscinosis (Tamaki et al., 2009) and MLD (Biffi et al., 2004; Givogri et al., 2006). The substantial attenuation of the neuropathology in the CNS of murine models should be reproduced in a larger brain before moving this therapeutic strategy into the clinic. In addition, a well-characterized and non-tumorigenic human-derived source of stem cells will be needed.

In general, several factors, including age at diagnosis, and HSCT, the clinical status, levels of chimerism, and specific genotype can also be determinants of the final clinical outcome for each patient undergoing this therapeutic modality (Biffi, 2017b). However, mortality has been reduced to approximately 10%, with variable rates for different lysosomal disorders (Prasad and Kurtzberg, 2008; Biffi, 2017b).

### CSF-Delivery of ERT Agents

Currently, ERT is considered an effective treatment for non-neurological manifestations of LSDs. It is conceivable that, to circumvent the BBB, ERT agents directly delivered into the cerebrospinal fluid (CSF) can achieve the neural cells. Several routes can be used, including intracerebroventricular (ICV) injection into the lateral ventricle (through a catheter/reservoir) or intrathecal (IT) injection into the lumbar spine or subarachnoid space at the cisterna magna lumbar puncture or an IT drug-delivery device. The first ERT agents to be CSF injected were laronidase (Aldurazyme) in a patient with MPS I- with cervical spinal cord compression (SCC), and unable to undergo necessary neurosurgery procedures (Munoz-Rojas et al., 2008). Another patient received the galsulfase ERT agent for MPS-VI (Bernal-Bayard et al., 2010). The IT-administered ERT was well-tolerated and safe, and some improvement in symptoms of SCC was observed (Klionsky et al., 2016). Later on, three-phase 1/2 studies investigating the safety (as primary outcome) and efficacy of CSF-delivery through IT injections for MPS-I, MPS-II, and MPS-III were performed (Klionsky et al., 2016; Muenzer et al., 2016; Nestrasil et al., 2017). One patient with attenuated MPS-I patient after receiving IT ERT showed signs of neurological benefits, improving brain structure, and reversing cognitive decline (Nestrasil et al., 2017). In addition, IT ERT was also assessed in conjunction with HSCT. Twenty-four patients received over 1–2 min in 4 mL of Elliott's B® solution containing recombinant ERT agent iduronidase (0.05 mg/kg) at four discrete time points: 8–12 weeks before HSCT, 2 weeks before HSCT, 100 days after HSCT, and 6 months after HSCT (Eisengart et al., 2019). All of the patients who demonstrated increases in IQ 2 years after treatment had at least a 50% reduction in CSF glycosaminoglycan non-reducing ends across the four-timepoints (Eisengart et al., 2019). The most notable limitation of this study is a lack of a comparison CSF from a group that received only intravenous ERT and HCT. In 2017, cerliponase alfa (Brineura®) was approved-based on the clinical trials showing to slow loss of ability to walk or crawl (ambulation) in symptomatic pediatric patients 3 years of age and older with late infantile neuronal ceroid lipofuscinosis type 2 (CLN2), also known as tripeptidyl peptidase 1 (TPP1) deficiency. In a clinical study of 24 patients with CLN2 receiving an intraventricular infusion of cerliponase alfa, the slope of decline in motor and language function was less than the one observed in the historical controls. Serious adverse events included the failure of the intraventricular device and device-related infections (Schulz et al., 2018).

In terms of immunogenicity, in the majority of patients who had intravenous ERT at least 6 months preceding the IT administration, no immune response or antibody was present in the CSF (Klionsky et al., 2016)]. The patients who developed antibodies against the ERT agents in the CSF did before the IT-ERT administration, and no safety issues were a concern. It is important to point out that significant ERT agent leakage occurs to the systemic circulation during IT-ERT administrations (Chung et al., 2017). For this reason, CSF-administered ERT agents can eventually result in systemic effects in non-CNS areas of patients receiving only this route as ERT administration.

### Gene-Therapy

The LSDs, as single-gene disorders with well-characterized biochemical and clinical phenotypes, are ideal candidates for gene therapy approaches. Also, the critical threshold of 10–15% of residual enzymatic activity for most lysosomal enzymes, below which no disease is manifested (Conzelmann and Sandhoff, 1983; Sandhoff and Conzelmann, 1984), has shown that only a small percentage of correction is required to reinstate the physiological turnover of the primary substrate and shift the cell homeostasis (Conzelmann and Sandhoff, 1991; Schueler et al., 2004). The gene therapy approach can be accomplished in two general ways, *in vivo* and *ex vivo*, which are both discussed below.

#### *In vivo* Gene Therapy

The gene therapy (GT) approach has made significant advances in several animal models of LSDs (Platt, 2018). Based on the encouraging pre-clinical studies in animal models, early phase clinical trials have started and shown positive results. A number of approaches have been developed to administer GT agents to target the CNS. Most of them are based on the direct administration of recombinant adeno-associated vectors (rAAVs) administered systemically (mostly intravenously) or locally injected (CNS). The rAAVs are derived from small, non-enveloped, and non-integrating AAVs. The transduction efficiency and safety characteristics make the rAAVs the ideal candidate for gene delivery (Bey et al., 2017). The rAAV serotypes traditionally used in gene-transferring into CNS are the 1, 2, 5, 8, 9, and the recombinant human (rh)10. Usually, rAAV-mediated gene transfer in the brain efficiently targets neurons and scarcely astrocytes, oligodendrocytes, and microglia (Hocquemiller et al., 2016). Specific rAAV serotypes, including AAV9 and 10, can cross the BBB, allowing transduction of the CNS after systemic administration (Foust et al., 2009; Hocquemiller et al., 2016). As issues raised on the CSF-injected ERT, because of leakage, local rAAV administration into the CNS results in transduction of non-target tissues and peripheral transgene expression, particularly in the liver (Hocquemiller et al., 2016). The leakage may trigger immune responses and can mitigate the efficacy of the *in vivo* GT. Transient immunosuppression as induction of tolerance has been developed (Colella et al., 2018; Keeler et al., 2019). In particular, relevant results have been obtained with strategies inducing liver-mediated tolerance or neonatal AAV-mediated systemic expression of a therapeutic protein before CNS-directed *in vivo* GT (Hinderer et al., 2015). Based on this and other preclinical studies (Fraldi et al., 2007), a phase I/II clinical trial was initiated in patients affected by MPS type IIIA. In this study, four children aged between 2 years 8 months and 6 years received intracerebral injections of an adeno-associated virus (AAV) vector carrying the complementary DNA encoding sulfamidase and the gene for the sulfatase-modifying factor *SUMF1* (Tardieu et al., 2014). The therapeutic vector was administered to the brain by stereotaxic surgery. The immunosuppressive regimen given 2 weeks before the procedure was generally well-tolerated. In two patients, the brain magnetic resonance imaging (MRI) showed brain size and neuropsychological tests were performed. The brain atrophy seemed to be stable, but tended to increase in the other two individuals. In three other patients, moderate improvements in neurocognitive areas, including behavior, attention, and sleep, were noted in neuropsychological evaluations (Tardieu et al., 2014; Marco et al., 2019). Intracerebral injections of adeno-associated viruses carrying a therapeutic gene have also been performed or are under evaluation in other LSDs and neurodegenerative diseases (Hocquemiller et al., 2016).

#### *Ex vivo* Therapy

As we described earlier, the BBB prevents circulating molecules, either small or macromolecules, from entering the CNS (Pardridge, 2007b). Hematopoietic cells permeate the BBB, where they subsequently differentiate into microglia. The HSCT in neurological LSDs may be beneficial but is associated with significant risks of GVHD host and other complications that range from 8 to 10%, even in the most advanced centers (Graf, 2017; Tan et al., 2019). In this setting, to circumvent the risks associated with the allogenic HSCT, autologous HSCs becomes attractive as it opens the opportunity to genetically modify the patient's HSCs to express the deficient lysosomal protein. The lentiviral vectors (LVs) showed strong safety and the most efficient tool to deliver genes to HSCs (Magrin et al., 2019). The lentiviral genome is stably integrated into the host cell genome after transduction, allowing a long-term and stable transgene expression over time and in the differentiated progeny of the original transduced HSPCs (Naldini, 2019). In general, to inject the lenti-transduced HSCs, a partial or full myeloablative bone marrow will be required to allow “space” and promote the novel HSCs engraftment (Biffi, 2017b; Naldini, 2019). One successful *ex vivo* gene therapy targeting CNS has been reported in MLD, an LSD caused by arylsulfatase A (ASA) deficiency and manifested by a severely progressive demyelination of CNS and peripheral nervous system. Based on previous studies (Lattanzi et al., 2010; Meneghini et al., 2016), nine children asymptomatically diagnosed with MLD underwent haemopoietic stem-cell gene therapy (HSC-GT) in a non-randomized, open-label, single-arm phase 1/2 trial with 3-years follow-up (Sessa et al., 2016). Initial data of some of the first subjects enrolled were published earlier (Biffi et al., 2013). After reinfusion of the genetically modified cells, high enzyme activity was found throughout haematopoietic lineages and in the cerebrospinal fluid. Eight patients, seven of whom received treatment when presymptomatic, had prevention of disease onset or halted disease progression as per clinical and instrumental assessment, compared with historical untreated control patients with early-onset disease. Gross Motor Function Measures scores for six patients up to the last follow-up showed that gross motor performance was similar to that of normally developing children. The extent of benefit appeared to be influenced by the interval between HSC-GT and the expected time of disease onset (Sessa et al., 2016). The *ad-hoc* results showed evidence of the safety and therapeutic benefit of *ex vivo* gene therapy for a neurological LSD when administered at presymptomatic or very early-symptomatic disease stages. Despite these promising results, the long-term safety and efficacy of the approach are needed to assure options for patients affected MLD and other LSDs (Sessa et al., 2016).

### Nano-Vesicle-Based Delivery of Therapies for LSDs

**Figure 2:**
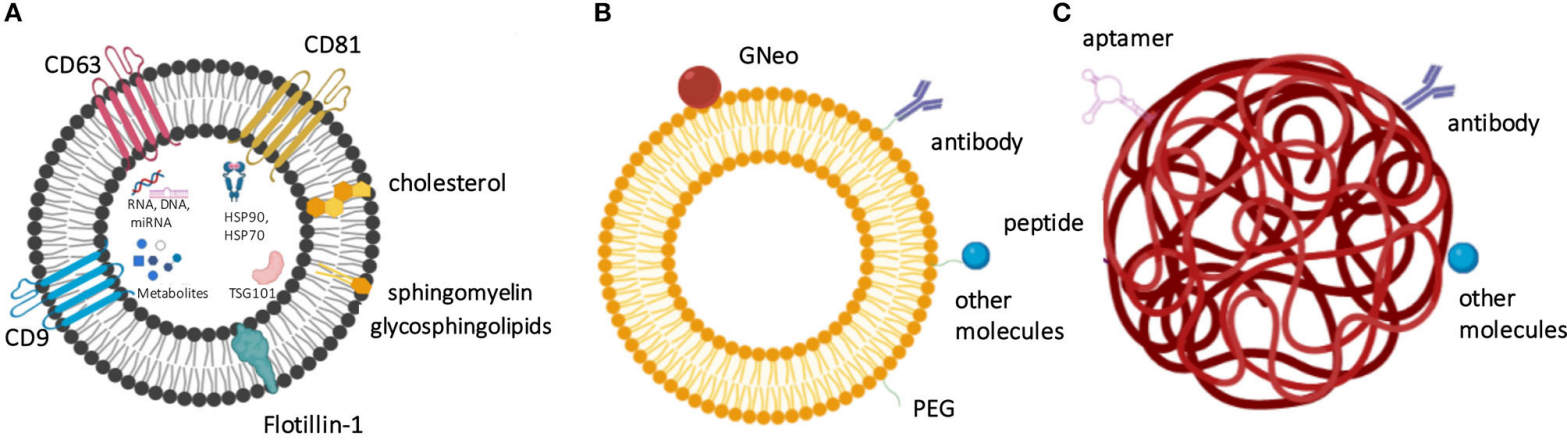
The nanocarrier-based delivery systems proposed for the treatment of lysosomal storage diseases include exosomes **(A)**, liposomes **(B)**, and nanoparticles **(C)**. **(A)** Exosomes as physiological extracellular vesicles produced by cells and contain specific protein- and lipid-based markers and also carry various metabolites, nucleic acids and proteins as cargo. **(B)** Liposome surface can be derivatized to contain targeting molecules, such as antibodies, GNeo, and other molecules, which are often attached to the liposomes after prior coating with polyethylene glycol (PEG). **(C)** Nanoparticles, such as here shown polymeric nanoparticle can be modified by the presence of peptides, aptamers, antibodies, and other molecules to increase their targeting efficiency.

Since the BBB forbids the translocation of large proteins into the CNS, only certain small low-molecular-weight or lipophilic molecules can pass through this natural barrier. Several types of nanovesicle-based delivery technologies have been designed to transport therapeutic agents to CNS to allow BBB delivery and membrane permeability. Amongst these approaches, polymeric nanoparticles (NPs), lipid NPs, liposomes, as well as extracellular vesicles (EVs), such as exosomes, produced by cells can be used to transport bioactive enzymes and other types of therapeutic molecules to CNS (Figure 2, Table 3). The specific characteristics that allow delivery through BBB are the size of vesicles/ NPs, their lipophilic composition, and the ability to carry a high payload of the chosen therapeutic agent. The modifications of these nanomaterials with specific ligands can open the possibility to target the nanocarriers to a specific tissue, cell, and even an organelle. In addition, these nanosystems offer a protective niche for the delivered cargo, due to increased physicochemical and biological stability of encapsulated and carried products. Intravenously injected enzymes avoid being exposed to blood and reach the body organs more efficiently if these molecules can be protected by the nanocarriers. Moreover, the delivery of therapeutic molecules to organs of interest in a more targeted manner can make the delivery more effective and control the half-life of the delivered enzymes. The qualities of these nanosystems also provide improved bioavailability, and many of these vehicles of delivery are non-toxic, biocompatible, and biodegradable (Onaca-Fischer et al., 2012; Pirooznia et al., 2012; Del Grosso et al., 2019).

**Table 3:** Vesicle-based delivery systems of therapeutic molecules for the treatment of LSDs.

**Vesicle type**	**Modification**	**Carried enzymatic or other cargo**	**Condition associated with enzyme deficiency**	**Type of study**	**References**
**EXOSOMES**					
HEK293-derived exosomes	Vesicular stomatitis virus glycoprotein for the improved le of carried cargo protein	β-glucocerebrosidase	Gaucher disease	*In vitro* study	Do et al., 2019
macrophage-derived EVs		tripeptidyl peptidase-1	Neuronal Ceroid Lipofuscinoses (NCL)-2, Batten disease	*In vitro* and *In vivo* study	Haney et al., 2019
**LIPOSOMES**					
liposome		β-galactosidase	GM1 gangliosidosis	*In vivo* study	Gregoriadis, 1978
liposome		α-mannosidase	Mannosidosis	*In vivo* study	Patel and Ryman, 1974
liposome		neuraminidase	Mucolipidosis type I	*In vivo* study	Gregoriadis et al., 1974
liposome		β-galactosidase	Krabbe disease	*In vivo* study	Umezawa et al., 1985
nanoliposomes	Functionalized with Arginine-Glycine-Aspartic acid (RGD) peptides for improved cellular uptake	α-galactosidase	Fabry disease	*In vivo* study	Cabrera et al., 2016
GNeo-liposomes	Guanidinylated neomycin (GNeo) transporter for enhanced delivery to lysososmes	α-l-iduronidase	Mucopolysaccharidosis type I	*In vitro* study	Hamill et al., 2017
liposomes	Lysosomotropic octadecyl-rhodamine B (Rh) for enhanced delivery to lysososmes	Glucocerebroside velaglucerase alfa	Gaucher disease	*In vitro* study	Koshkaryev et al., 2011
**NANOPARTICLES**					
PLGA acidic NPs	Acidic NPs for re-acidification of the defective lysosomes		glucocerebrosidase-mutant cells, PD	*In vitro* and *in vivo* study	Bourdenx et al., 2016
NPs	saposin C for the protection of biologically active cargo	acid b-glucosidase	Pompe disease	*In vivo* study	Sun et al., 2020
PLGA NPs	Ang2- (Angiopep-2), g7, and Tf2 (transferrin binding)-functionalized NPs to improve the delivery to brain	galactosylceramidase	Krabbe disease	*In vivo* study	Del Grosso et al., 2019
polystyrene particle	Coupled to anti-ICAM-1 antibody for an improved targeting to organs	α-galactosidase	Fabry disease	*In vivo* and *in vitro* study	Hsu et al., 2014, Hsu et al., 2011
polystyrene particle	Coupled to anti-ICAM-1 antibody for an improved targeting to organs	acid α-glucosidase	Pompe disease	*In vivo* study	Hsu et al., 2012
NPs	Coupled to anti-ICAM-1 antibody for an improved targeting to organs	acid sphingomyelinase	Acid sphingomyelinase deficiency	*In vitro* study	Muro et al., 2006
PLGA NPs	7-aminoacid glycopeptide for increased targeting of NPs	Albumin was used as a model	NPs were tested in MPS-I and II models	*In vivo* and *in vitro* study	Salvalaio et al., 2016
Protein-based NPs	Human serum albumin (HSA) and 30 Kc19 silkworm proteins were used to make the NPs to enhance cellular uptake	α-galactosidase	Fabry disease	*In vitro* study	Lee et al., 2016
trimethyl chitosan-based polyelectrolyte complex-based nanocarriers	respond to low pH by the triggered release of the loaded protein for controlled release, Atto 647 N modification	α -galactosidase	Fabry disease	*In vitro* study	Giannotti et al., 2011
quantum dots	Guanidinylated neomycin (GNeo) for improved cell binding	β-glucuronidase or α-iduronidase	MPS-VII and MPS-I	*In vitro* study	Sarrazin et al., 2010

*ICAM-1, intercellular Adhesion Molecule 1; MPS, mucopolysaccharidosis; PD, Parkinson disease*.

#### The EV-Mediated Delivery of Therapied to Target CNS

##### The heterogeneity of EVs

All eukaryotic cells secrete extracellular vesicles (EVs), which vary in function and characteristics, but they all carry specific molecules from the cell of origin to the target cells, thus performing essential roles in the cell-to-cell communication and homeostasis. EVs are a heterogeneous population of vesicles that vary in size, biogenesis, and protein markers (Raposo and Stoorvogel, 2013). The subpopulation of EVs include exosomes, microvesicles (MVs), apoptotic bodies (APOs), oncosomes (Di Vizio et al., 2009; Minciacchi et al., 2015), and exomeres (Nolte-'T Hoen et al., 2012). In particular, exosomes are small EVs, which have a size a range of 30–120 nm (Théry et al., 2002), contain tetraspanin-based biomarkers CD9, CD63, or CD81 (Raposo and Stoorvogel, 2013), and have unique biogenesis originating in the endosomal pathway (Raposo and Stoorvogel, 2013). Exosomes are capable of carrying and transmitting protein and RNA cargo, but also specific lipids and small molecules (Rajendran et al., 2006; Alvarez-Erviti et al., 2011a; Noerholm et al., 2012). This exosomal cargo can be subsequently transmitted to other cells. Because exosomes travel in physiological fluids, these vesicles can reach cells in distant organs to transmit their contents from the cells of origin (Alvarez-Erviti et al., 2011b). Since exosomes are formed by all known cell types, not surprisingly, they are also present in the CNS. Within the brain, exosomes have specific functions regulating the interactions between cells, such as neurons and glial cells, for instance by mediating and facilitating the delivery of bioactive molecules from one cell to another (Frühbeis et al., 2012; Zhang and Yang, 2018).

##### Brain-homing signals for exosomes

Although exosomes contain some molecules on their surface that allow them to reach specific organs, engineered exosomes containing modified membrane *decorations* that result in improved targeted delivery. One of the ways by which exosomes can be engineered to possess increased targeting capabilities is by a fusion of targeting peptides to the extracellular region of proteins displayed on naïve exosomes. One of such proteins with an extracellular *N*-terminus is Lamp2b (Alvarez-Erviti et al., 2011b), which is abundantly present on exosomal membranes. Lamp2b molecule can then fused with a peptide utilized for the targeting of specific organs (Alvarez-Erviti et al., 2011b). For the CNS-specific deliveries, rabies viral glycoprotein (RVG) peptide (YTIWMPENPRPGTPCDIFTNSRGKRASNG) can be used, which specifically binds to the acetylcholine receptor 3. Dendritic cells-derived exosomes engineered to express an exosomal membrane protein, Lamp2b, which was fused to the neuron-specific RVG (rabies virus glycoprotein) peptide were used to deliver exosomes to the brain by intravenous injections. These exosomes containing this siRNA cargo were introduced by electroporation. By using this model, exosomes were able to deliver the cargo to neurons, microglia, and oligodendrocytes in the brain, where the carried siRNA resulted in a gene knockdown (Alvarez-Erviti et al., 2011b). The RVG peptide was also used by other groups for exosome homing to the brain (Yang et al., 2017). Another type of peptide that can be fused with Lamp2b to target exosomes to the brain is T7 peptide. Antisense miRNA oligonucleotides against miR-21 (AMO-21) with potential for the treatment of glioblastoma were delivered by exosomes decorated with Lamp2b-fused T7 peptide since the transferrin receptor is overexpressed on the surface of glioblastoma cells. T7-exosomes had a higher delivery efficiency to glioblastoma cells in comparison to the unmodified exosomes or RVG-decorated exosomes *in vitro*. Similar observations were obtained *in vivo*, where the delivery of AMO-21 by exosomes via an intravenous injection led to a reduction in the miR-21 levels, followed by the reduction of tumor size (Kim et al., 2020). Finally, muscle-specific peptide (MSP) identified by *in vivo* phage display (ASSLNIA) can also be fused with Lamp2b for muscle-specific delivery of exosomes (Alvarez-Erviti et al., 2011b).

##### Exploring Therapeutic EVs to Treat Disorders Affecting CNS

Although exosomes were evaluated in terms of the therapeutic function in such CNS conditions as glioblastoma (Kim et al., 2020), these and other natural nanovesicles have not been extensively characterized as delivery vehicles of therapeutic agents for LSD. A single study focused on exosome-based delivery of a lysosomal enzyme for LSD treatment, in which lysosomal β-glucocerebrosidase (GBA) was fused to a protein that anchored it to the exosomes, termed vesicular stomatitis virus glycoprotein (VSVG), without altering the protein function, but significantly increasing the levels of β-glucocerebrosidase. This is an evidence that the lysosomal the enzyme payloads within exosomes are active. The β-glucocerebrosidase enclosed within these HEK293-derived exosomes was taken up by HEK293 recipient cells by the endocytosis pathway. Finally, such obtained exosomes were explicitly targeted to endocytic compartments and increased GBA activity in the recipient cells (Do et al., 2019).

Macrophage-derived EVs were also used for brain delivery of a soluble lysosomal enzyme tripeptidyl peptidase-1, TPP1, for the treatment of Neuronal Ceroid Lipofuscinoses 2 (CLN2) or Batten disease (Haney et al., 2019). Because macrophages are often associated with inflamed tissue, macrophage derived EVs were hypothesized to interact with inflamed tissues for enhanced targeted delivery of the therapeutic protein. In this study, TPP1-encoding plasmid DNA was transfected into macrophages, or TPP1 protein was packed into the empty EVs by sonication or permeabilization with saponin, and all these methods yielded proper incorporation of functional TPP1 into the vesicles. EVs also increased the stability of TPP1 and were able to deliver the protein to the recipient cells, where over 70% of the protein was localized to lysosomes. TPP1-containing EVs were also administered intraperitoneally and led to the accumulation of TPP1-EVs in brain tissue, improving the lifespan of the late-infantile neuronal ceroid lipofuscinosis mouse (Haney et al., 2019).

Finally, companies such as Evox Therapeutics have an interest in applying exosome-based deliveries for the improved treatment of Niemann-Pick disease type C (Zipkin, 2019). The exosomes produced by Evox Therapeutics carry a copy of the NPC protein to the neural cells by using surface molecules that bind certain, yet undisclosed, ligands (Hean et al., 2019). The EV-based therapies are promising delivery systems for the proteins, which carry large payloads of cargo, and also vectors which are used for gene therapy-based therapeutic approaches.

#### Liposome-Based Delivery of LSD Therapeutics

Liposomal drug delivery systems composed of biocompatible nanomaterials enhance the efficacy of ERT since these nanostructures encapsulate and protect the enzymes in biodegradable micelles. Liposomes are spherical vesicles that contain one or more lipid bilayers, and their hydrophilic character is ideal for efficient drug delivery. The bilayer components affect the properties of liposomes, including the rigidity, charge, and fluidity of the bilayer. Unsaturated phosphatidylcholine lipids obtained from natural sources can be used to manufacture liposomes, but these species of lipids increase the permeability and negatively affect the stability of these vesicles. On the other hand, saturated phospholipids containing longer acyl chains are more rigid and impermeable (Akbarzadeh et al., 2013). ERT utilizing liposomes that contain enzymes which are deficient in LDS were already proposed in the 1970s and 1980s (Patel and Ryman, 1974, Gregoriadis et al., 1974, Steger and Desnick, 1977, Gregoriadis, 1978, Umezawa et al., 1985). However, in these early studies, liposomes often failed to specifically localize the enzyme of interest to the brain (Umezawa et al., 1985). Since then, considerable effort has been made to improve the targeted delivery of therapeutic proteins to CNS by liposomes.

First, liposomes can be modified to contain homing signals to enhance liposome-mediated transport to specific organs. Immunoliposomes—or antibody-directed liposomes—are sterically stabilized with PEG, which is conjugated to a linker lipid, allowing for conjugation of thiolated antibody on the surface of the vesicle. The monoclonal antibodies are used as modifiers attached to the surface of such liposomes. Examples of antibodies that were previously used to direct the liposomes to the brain are the OX26 antibody recognizing the transferrin receptor, which increased the uptake of liposomes to rat brain and specifically to the microvascular endothelium that builds the blood-brain barrier (Huwyler et al., 1996).

Second, the liposome modification can increase the uptake of liposomes by cells. Here, modification of the liposomal surface with a peptide has enhanced the intracellular penetration of liposomes. Also, nanoliposomes functionalized with Arginine-Glycine-Aspartic acid (RGD) peptides containing α -galactosidase-a, had an enhanced enzymatic activity and improved intracellular penetration (Cabrera et al., 2016). Proteins such as apolipoprotein E can be added on the surface of liposomes for more efficient delivery of receptor-mediated uptake of the liposomes to the cells (Ansari et al., 1997).

To further improve the delivery of liposomes to lysosomes, guanidinylated neomycin (GNeo) transporter can be used. GNeo is capable of transport of bioactive cargo inside the cells, which can also be of high molecular weight. GNeo works by a process utilizing heparan sulfate proteoglycans in the cell surface. The synthesis of GNeo-lipids, which were then incorporated into the liposomes, termed GNeo-liposomes, or GNeosomes, was able to enhance the specific delivery of these particles to lysosomes, which encapsulated α-l-iduronidase (IDUA). These GNeosomes successfully restored the function in fibroblasts isolated from a patient that lacked this enzyme due to the lysosomal storage disorder mucopolysaccharidosis type I (MPS I) (Hamill et al., 2017). Finally, lysosomotropic octadecyl-rhodamine B (Rh) (Koshkaryev et al., 2011) was used to increase the delivery of the encapsulated glucocerebroside velaglucerase-α to the lysosomes in the Gaucher's fibroblasts, where a 68% increase in the targeted delivery was observed in contrast to liposomes that were not modified with the Rh (Thekkedath et al., 2013).

#### NP-Based Delivery of Therapeutic Molecules for the Treatment of LDS

Another particle type that can be used for the delivery is polymeric NP. Poly-(lactide-co-glycolide), PLGA is one of the preferred materials used for the production of NPs, which is a non-toxic material and thus offers increased biocompatibility (Patel et al., 2012). PLGA has been approved by the Food and Drug Administration for administration in humans. As an example, the catalytic activity of lipase was improved by immobilization of this enzyme onto a porous super magnetic polymeric microsphere carrier. The glycerol-oleic-acid-esterification activity of the immobilized lipase increased seven fold in comparison with the naked enzyme. Whereas, the activity normalized to the total protein content of the immobilized enzyme was improved by 200-fold, and the stability of the lipase was also improved by the immobilization of this enzyme to the NPs (Meng et al., 2013).

The targeting function of NPs can be enhanced by the NP modifications, where peptides, antibodies, and aptamers can be added to the surface of these structures. As an example, Ang2- (Angiopep-2) (Demeule et al., 2008), g7 (Tosi et al., 2011), and Tf2 (transferrin binding)-functionalized (Santi et al., 2017). PLGA NPs containing cross-linked enzyme aggregates were tested in the encapsulation efficiency, activity yield of encapsulated enzymes, and targeting to the brain if delivered intraperitoneally to a TWI murine model of Krabbe disease (Del Grosso et al., 2019). These NPs were able to promote recovery in the brain up to the control mice level. Specifically, the enzyme-delivering NPs lead to significant GALC activity in the organs, where both targeted and control NPs accumulated in the liver and kidneys 4 h after injection. However, only the targeted NPs were detected in the brain, displaying a GALC activity that was comparable to the activity of the heterozygous mice for GALC, which is identical to the wild-type mouse phenotype.

In contrast, the non-targeted NPs did not show any significant GALC activity increase in the brain tissues (Del Grosso et al., 2019).

Another modification of NPs is antibody coupling. One of the commonly targeted molecules is ICAM-1, which is an endothelial surface protein upregulated in many LSD pathologies, such as inflammation or metabolic imbalance (Springer, 1994). Polystyrene and PLGA polymer nanocarriers targeting ICAM-1, delivered by intravenous injection to mice, were reported not to cause lung injury or have other adverse effects, such as abnormal vascular permeability. These NPs were rapidly removed from the circulation and accumulated in various organs such as the kidney, heart, liver, spleen, or lung (Garnacho et al., 2008). Polystyrene particles coupled to an antibody specific to ICAM-1 were then used to improve targeting of α-galactosidase (αGal), which is an enzyme affected in the Fabry disease. While naked αGal intravenously delivered to animals preferentially remained in circulation and not up-taken by tissues and organs, αGal coupled to nanocarriers accumulated in the brain, kidneys, heart, liver, lungs, and spleen (Hsu et al., 2014). The nanocarriers also specifically were targeted to lysosomes and led to an enhanced globotriaosylceramide degradation, thus constituting possible improvement of drug delivery for Fabry disease (Hsu et al., 2011). The same ICAM-1 molecule was targeted for the delivery of ERT to treat Pompe disease caused by a deficiency of acid α-glucosidase (GAA). GAA was coupled to ~180 nm polystyrene bead-based nanocarriers coated with anti-ICAM-1, and this enhanced targeting of anti-ICAM/GAA NCs to organs in comparison to the naked enzyme delivery (Hsu et al., 2012). Anti-ICAM-1-coated nanocarriers have also been used to target recombinant proteins, such as human acid sphingomyelinase to ICAM-1-positive cells, including activated endothelial cells and Niemann–Pick disease patient fibroblasts. The sphingomyelinase delivery by nanocarriers was enabled by the CAM-mediated endocytosis pathway, which is distinct from the clathrin-dependent endocytosis. As a result, the bioactive proteins were successfully delivered to lysosomes, leading to a decreased accumulation of lysosomal lipids (Muro et al., 2006).

Peptides are another class of modifications that can be added to NPs to enhance their targeting to brain regions. A 7-aminoacid glycopeptide (g7) was used to decorate PLGA NPs, which were then tested in MPS I and MPS II models. The g7-modified PLGA NPs successfully delivered high molecular weight molecules across the BBB in a murine model, where a model molecule FITC-albumin was used as an example (Salvalaio et al., 2016).

Human serum albumin (HSA) and 30Kc19 silkworm protein were tested as an enhancement strategy for the delivery of α-galactosidase. HSA was used because this protein is an FDA-approved molecule, and it binds to receptors within the caveolae while the 30Kc19 silkworm protein enhances cellular uptake and stabilizes the cargo (Park et al., 2012, 2014). Using primary cultured fibroblast, 30Kc19-HSA NPs led to enhanced cellular uptake of the carried protein. The α-galactosidase carried by these NPs had improved globotriaosylceramide degradation in the fibroblasts (Lee et al., 2016).

Controlled release of proteins to the lysosomes can also be achieved by functional polyelectrolyte-based NPs, which respond to low pH by the triggered release of the loaded protein. In one study, trimethyl chitosan (TMC)-based polyelectrolyte complexes (PECs) nanocarriers were used to deliver α-Gal. These NPs released the enzyme at acidic pH, and upon their further functionalization with fluorescent group Atto 647N, PECs were shown to be further internalized by human endothelial cells and enriched in the lysosomes (Giannotti et al., 2011).

Apart from transferring molecules, NPs can be used to lower the pH within lysosomes. PLGA acidic NPs, which were transported to lysosomes, were able to restore impaired lysosomal function glucocerebrosidase-mutant cells by inducing re-acidification of the defective lysosomes. The same PLGA-acidic NP was localized to neurons following the intracerebral injection, where they were able to attenuate the neurodegeneration *in vivo* in the murine model of PD by rescuing the lysosomal dysfunction (Bourdenx et al., 2016).

Other modification to the nanovesicles include the addition of stabilization agents. Saposin C (SapC) is a lysosomal glycoprotein which naturally protects GCase from the degradation (Sun et al., 2003). The CNS-selective delivery system based on nanovesicles of saposin C (SapC) and dioleoylphosphatidylserine (DOPS) was used to deliver acid b-glucosidase (GCase) via the BBB. The enclosure of the enzyme within the nanovesicles improved the stability and activity of the GCase and mediated the uptake of the enzyme by a mannose receptor-independent pathway. SapC-DOPS-GCase-containing nanovesicles were able to penetrate through BBB via the surface phosphatidylserine. These vesicles also led to a reduction of GCase substrate levels, attenuating brain inflammation (Sun et al., 2020).

#### Quantum Dots

One more category of the nanocarriers worth mentioning are quantum dots, also known as “artificial atoms,” which are semiconductor-based nanocrystals in a size range of 2–10 nm. Guanidinylated neomycin (GNeo), which targets liposomes to lysosomes (Hamill et al., 2017), as described above, was also used to modify quantum dots, which were able to undergo endocytosis and translocate the lysosomes. The activated ester of GNeo was conjugated to β-glucuronidase or α–iduronidase enzymes, which did not affect enzymatic activity. This GNeo modification enabled efficient binding of the heparan sulfate on primary human fibroblast cells, where the GNeo quantum dots containing the enzyme led to a restoration of glycosaminoglycan turnover in the cells (Sarrazin et al., 2010).

## Conclusions and Future Directions

The mainstay therapy for LSDs, ERT, and current small molecules functioning as SRT and PC agents have a very limited biodistribution to CNS, PNS, and even other organs including bone, skeletal and cardiac muscle and connective tissues (Hollak and Wijburg, 2014). A summary of the current therapies targeting the CNS are described in Table 2. In the majority of LSDs, the predominant clinical manifestations are neurological in origin and associated with progressive morbidity. Therefore, there is an urgent and unmet need for therapies to the CNS, tackling the primary defect (enzyme deficiency/substrate build-up), and the multiple secondary pathogenic cascades (Pastores and Maegawa, 2013; Giugliani et al., 2018; Platt, 2018; Platt et al., 2018). Despite remarkable advances in the understanding of neuropathogenesis of these disorders, the CNS impairment remains the major cause of disabilities, hospitalizations, and the requirement of prolonged and complex care for the affected patients. Cognizance of this unmet therapeutic need and challenges imposed by the BBB, several therapeutic modalities have been investigated, including modified ERT agents, novel small molecules, HSCT, gene therapy and innovative therapeutic-CNS delivery strategies. The advances in combinatorial chemistry, nanomolecule and chemical synthesis allowed the expansion of the chemical space, resulting in novel and unique molecules capable of permeating the BBB and tackling the neurodegenerative processes in the CNS. A better understanding of exosomes and other nanovesicles as carriers of signaling metabolites will allow us to explore the therapeutic aspect of these nanovesicles (O'loughlin et al., 2012; Van Niel et al., 2018). The advances in, now “micro,” robotic technologies, high-resolution microscopy will offer multiple and concomitant readouts in high-through screening (HTS) assays, generating more disease-relevant and precise and “hits.”(O'loughlin et al., 2012; Van Niel et al., 2018). In addition, the “tissue-chips” or “microphysiological systems,” bioengineered microsystems capable of recreating aspects of human organ physiology and function, will accelerate the validation and prioritization of the small molecule “hits” against a specific target and its mutant variants (Low et al., 2020). With the advances of mass spectrometry and next-generation sequencing (NGS), allowing both newborn screen programs and early diagnosis, respectively, an increasing number of individuals are being diagnosed at early or presymptomatic disease stages. In this setting, diverse modalities and more tailored and individualized therapeutics will be required to treat and, eventually prevent, the lysosomal, cellular, and organ damages, often irreversible by the time of diagnosis. In sum, applying the basic understanding of the neuropathogenesis to develop safer and target therapies, the prevention of the LSD manifestations throughout the life span of early diagnosed individuals, who maybe “apparently healthy,” will be achieved.

## Author Contributions

GM and ME contributed to the design, writing, and review of the manuscript. All authors contributed to the article and approved the submitted version.

## Conflict of Interest

GM has received research funding from NINDS/NIH, U.S. Department of Defense, Pfizer Inc., Moderna Inc., Protalix Pharm. and also non-profit foundations as The Legacy of Angels Foundation. GM has received honorarium for consulting for Genzyme-Sanofi. The remaining author declares that the research was conducted in the absence of any commercial or financial relationships that could be construed as a potential conflict of interest.

## References

[B1] Abbott N. J. Patabendige A. A. Dolman D. E. Yusof S. R. Begley D. J. (2010). Structure and function of the blood-brain barrier. Neurobiol. Dis. 37, 13–25. 10.1016/j.nbd.2009.07.030 19664713

[B2] Acosta W. Cramer C. L. (2020). Targeting macromolecules to CNS and other hard-to-treat organs using lectin-mediated delivery. Int. J. Mol. Sci. 21:971. 10.3390/ijms21030971 32024082 PMC7037663

[B3] Aflaki E. Borger D. K. Moaven N. Stubblefield B. K. Rogers S. A. Patnaik S.. (2016). A new glucocerebrosidase chaperone reduces alpha-synuclein and glycolipid levels in iPSC-derived dopaminergic neurons from patients with gaucher disease and parkinsonism. J. Neurosci. 36, 7441–7452. 10.1523/JNEUROSCI.0636-16.2016 27413154 PMC4945664

[B4] Akbarzadeh A. Rezaei-Sadabady R. Davaran S. Joo S. W. Zarghami N. Hanifehpour Y. (2013). Liposome: classification, preparation, and applications. Nanoscale Res. Lett. 8:102 10.1186/1556-276X-8-102 23432972 PMC3599573

[B5] Aldenhoven M. Wynn R. F. Orchard P. J. O'meara A. Veys P. Fischer A.. (2015). Long-term outcome of Hurler syndrome patients after hematopoietic cell transplantation: an international multicenter study. Blood 125, 2164–2172. 10.1182/blood-2014-11-608075 25624320

[B6] Alderson L. M. Joksaite S. X. Kemp J. Main E. Watson T. Platt F. M.. (2019). Age-related gait standards for healthy children and young people: the GOS-ICH paediatric gait centiles. Arch. Dis. Child. 104, 755–760. 10.1136/archdischild-2018-316311 30910816

[B7] Allewelt H. Taskindoust M. Troy J. Page K. Wood S. Parikh S.. (2018). Long-term functional outcomes after hematopoietic stem cell transplant for early infantile krabbe disease. Biol. Blood Marrow Transplant. 24, 2233–2238. 10.1016/j.bbmt.2018.06.020 29933067

[B8] Alvarez-Erviti L. Seow Y. Schapira A. H. Gardiner C. Sargent I. L. Wood M. J.. (2011a). Lysosomal dysfunction increases exosome-mediated alpha-synuclein release and transmission. Neurobiol. Dis. 42, 360–367. 10.1016/j.nbd.2011.01.029 21303699 PMC3107939

[B9] Alvarez-Erviti L. Seow Y. Yin H. Betts C. Lakhal S. Wood M. J. (2011b). Delivery of siRNA to the mouse brain by systemic injection of targeted exosomes. Nat. Biotechnol. 29, 341–345. 10.1038/nbt.1807 21423189

[B10] Ansari N. H. He Q. Cook J. D. Wen J. Srivastava S. K. (1997). Delivery of liposome-sequestered hydrophobic proteins to lysosomes of normal and Batten disease cells. J. Neurosci. Res. 47, 341–347. 10.1002/(SICI)1097-4547(19970201)47:3<341::AID-JNR12>3.0.CO;2-4 9039656

[B11] Balch W. E. Morimoto R. I. Dillin A. Kelly J. W. (2008). Adapting proteostasis for disease intervention. Science 319, 916–919. 10.1126/science.1141448 18276881

[B12] Ballen K. K. Gluckman E. Broxmeyer H. E. (2013). Umbilical cord blood transplantation: the first 25 years and beyond. Blood 122, 491–498. 10.1182/blood-2013-02-453175 23673863 PMC3952633

[B13] Barker J. N. Weisdorf D. J. Wagner J. E. (2001). Creation of a double chimera after the transplantation of umbilical-cord blood from two partially matched unrelated donors. N. Engl. J. Med. 344, 1870–1871. 10.1056/NEJM200106143442417 11407361

[B14] Begley D. J. Pontikis C. C. Scarpa M. (2008). Lysosomal storage diseases and the blood-brain barrier. Curr. Pharm. Des. 14, 1566–1580. 10.2174/138161208784705504 18673198

[B15] Bernal-Bayard J. Cardenal-Muñoz E. Ramos-Morales F. (2010). The Salmonella type III secretion effector, salmonella leucine-rich repeat protein (SlrP), targets the human chaperone ERdj3. J. Biol. Chem. 285, 16360–16368. 10.1074/jbc.M110.100669 20335166 PMC2871503

[B16] Bey K. Ciron C. Dubreil L. Deniaud J. Ledevin M. Cristini J.. (2017). Efficient CNS targeting in adult mice by intrathecal infusion of single-stranded AAV9-GFP for gene therapy of neurological disorders. Gene Ther. 24, 325–332. 10.1038/gt.2017.18 28425480

[B17] Bhattacharya A. Qi L. (2019). ER-associated degradation in health and disease - from substrate to organism. J. Cell Sci. 132:jcs.232850. 10.1242/jcs.232850 31792042 PMC6918741

[B18] Bidou L. Allamand V. Rousset J. P. Namy O. (2012). Sense from nonsense: therapies for premature stop codon diseases. Trends Mol. Med. 18, 679–688. 10.1016/j.molmed.2012.09.008 23083810

[B19] Biffi A. (2017a). Hematopoietic gene therapies for metabolic and neurologic diseases. Hematol. Oncol. Clin. North Am. 31, 869–881. 10.1016/j.hoc.2017.06.004 28895853

[B20] Biffi A. (2017b). Hematopoietic stem cell gene therapy for storage disease: current and new indications. Mol. Ther. 25, 1155–1162. 10.1016/j.ymthe.2017.03.025 28389320 PMC5417839

[B21] Biffi A. de Palma M. Quattrini A. Del Carro U. Amadio S. Visigalli I.. (2004). Correction of metachromatic leukodystrophy in the mouse model by transplantation of genetically modified hematopoietic stem cells. J. Clin. Invest. 113, 1118–1129. 10.1172/JCI200419205 15085191 PMC385395

[B22] Biffi A. Montini E. Lorioli L. Cesani M. Fumagalli F. Plati T.. (2013). Lentiviral hematopoietic stem cell gene therapy benefits metachromatic leukodystrophy. Science 341:1233158. 10.1126/science.1233158 23845948

[B23] Boado R. J. Hui E. K. Lu J. Z. Pardridge W. M. (2013). IgG-enzyme fusion protein: pharmacokinetics and anti-drug antibody response in rhesus monkeys. Bioconjug. Chem. 24, 97–104. 10.1021/bc3005123 23249376 PMC3549264

[B24] Boado R. J. Zhang Y. Zhang Y. Xia C. F. Wang Y. Pardridge W. M. (2008). Genetic engineering of a lysosomal enzyme fusion protein for targeted delivery across the human blood-brain barrier. Biotechnol. Bioeng. 99, 475–484. 10.1002/bit.21602 17680664

[B25] Boelens J. J. Orchard P. J. Wynn R. F. (2014). Transplantation in inborn errors of metabolism: current considerations and future perspectives. Br. J. Haematol. 167, 293–303. 10.1111/bjh.13059 25074667

[B26] Borgwardt L. Guffon N. Amraoui Y. Dali C. I. De Meirleir L. Gil-Campos M.. (2018). Efficacy and safety of Velmanase alfa in the treatment of patients with alpha-mannosidosis: results from the core and extension phase analysis of a phase III multicentre, double-blind, randomised, placebo-controlled trial. J. Inherit. Metab. Dis. 41, 1215–1223. 10.1007/s10545-018-0185-0 29846843 PMC6326984

[B27] Bourdenx M. Daniel J. Genin E. Soria F. N. Blanchard-Desce M. Bezard E.. (2016). Nanoparticles restore lysosomal acidification defects: implications for Parkinson and other lysosomal-related diseases. Autophagy 12, 472–483. 10.1080/15548627.2015.1136769 26761717 PMC4835967

[B28] Cabrera I. Abasolo I. Corchero J. L. Elizondo E. Gil P. R. Moreno E.. (2016). α-galactosidase-a loaded-nanoliposomes with enhanced enzymatic activity and intracellular penetration. Adv. Healthc. Mater. 5, 829–840. 10.1002/adhm.201500746 26890358

[B29] Camargo F. Erickson R. P. Garver W. S. Hossain G. S. Carbone P. N. Heidenreich R. A.. (2001). Cyclodextrins in the treatment of a mouse model of Niemann-Pick C disease. Life Sci. 70, 131–142. 10.1016/S0024-3205(01)01384-4 11787939

[B30] Capablo J. L. Franco R. de Cabezon A. S. Alfonso P. Pocovi M. Giraldo P. (2007). Neurologic improvement in a type 3 Gaucher disease patient treated with imiglucerase/miglustat combination. Epilepsia 48, 1406–1408. 10.1111/j.1528-1167.2007.01074.x 17433057

[B31] Cartier N. Aubourg P. (2008). Hematopoietic stem cell gene therapy in Hurler syndrome, globoid cell leukodystrophy, metachromatic leukodystrophy and X-adrenoleukodystrophy. Curr. Opin. Mol. Ther. 10, 471–478. 18830923

[B32] Charkhand B. Scantlebury M. H. Narita A. Zimran A. Al-Hertani W. (2019). Effect of Ambroxol chaperone therapy on Glucosylsphingosine (Lyso-Gb1) levels in two Canadian patients with type 3 Gaucher disease. Mol. Genet. Metab. Rep. 20:100476. 10.1016/j.ymgmr.2019.100476 31467847 PMC6713848

[B33] Chung J. K. Pan L. Palmieri K. Youssef A. S. Mccauley T. G. (2017). Whole body and CNS biodistribution of rhHNS in cynomolgus monkeys after intrathecal lumbar administration: treatment implications for patients with MPS IIIA. Int. J. Mol. Sci. 18:2594. 10.3390/ijms18122594 29194406 PMC5751197

[B34] Clarke J. T. Mahuran D. J. Sathe S. Kolodny E. H. Rigat B. A. Raiman J. A.. (2011). An open-label Phase I/II clinical trial of pyrimethamine for the treatment of patients affected with chronic GM2 gangliosidosis (Tay-Sachs or Sandhoff variants). Mol. Genet. Metab. 102, 6–12. 10.1016/j.ymgme.2010.09.004 20926324 PMC3019177

[B35] Clarke L. A. Atherton A. M. Burton B. K. Day-Salvatore D. L. Kaplan P. Leslie N. D.. (2017). Mucopolysaccharidosis type I newborn screening: best practices for diagnosis and management. J. Pediatr. 182, 363–370. 10.1016/j.jpeds.2016.11.036 27939258

[B36] Colella P. Ronzitti G. Mingozzi F. (2018). Emerging issues in AAV-mediated *in vivo* gene therapy. Mol. Ther. Methods Clin. Dev. 8, 87–104. 10.1016/j.omtm.2017.11.007 29326962 PMC5758940

[B37] Condori J. Acosta W. Ayala J. Katta V. Flory A. Martin R.. (2016). Enzyme replacement for GM1-gangliosidosis: uptake, lysosomal activation, and cellular disease correction using a novel beta-galactosidase:RTB lectin fusion. Mol. Genet. Metab. 117, 199–209. 10.1016/j.ymgme.2015.12.002 26766614 PMC6116835

[B38] Conzelmann E. Sandhoff K. (1983). Partial enzyme deficiencies: residual activities and the development of neurological disorders. Dev. Neurosci. 6, 58–71. 10.1159/000112332 6421563

[B39] Conzelmann E. Sandhoff K. (1991). Biochemical basis of late-onset neurolipidoses. Dev. Neurosci. 13, 197–204. 10.1159/000112160 1817024

[B40] Corraliza-Gomez M. Sanchez D. Ganfornina M. D. (2019). Lipid-binding proteins in brain health and disease. Front. Neurol 10:1152. 10.3389/fneur.2019.01152 31787919 PMC6854030

[B41] Cox T. Lachmann R. Hollak C. Aerts J. Van Weely S. Hrebicek M.. (2000). Novel oral treatment of Gaucher's disease with N-butyldeoxynojirimycin (OGT 918) to decrease substrate biosynthesis. Lancet 355, 1481–1485. 10.1016/S0140-6736(00)02161-9 10801168

[B42] Cox T. M. Drelichman G. Cravo R. Balwani M. Burrow T. A. Martins A. M.. (2015). Eliglustat compared with imiglucerase in patients with Gaucher's disease type 1 stabilised on enzyme replacement therapy: a phase 3, randomised, open-label, non-inferiority trial. Lancet 385, 2355–2362. 10.1016/S0140-6736(14)61841-9 25819691

[B43] Cutler C. Multani P. Robbins D. Kim H. T. Le T. Hoggatt J.. (2013). Prostaglandin-modulated umbilical cord blood hematopoietic stem cell transplantation. Blood 122, 3074–3081. 10.1182/blood-2013-05-503177 23996087 PMC3811179

[B44] Daneman R. Prat A. (2015). The blood-brain barrier. Cold Spring Harb. Perspect. Biol 7:a020412. 10.1101/cshperspect.a020412 25561720 PMC4292164

[B45] Davidson C. D. Ali N. F. Micsenyi M. C. Stephney G. Renault S. Dobrenis K.. (2009). Chronic cyclodextrin treatment of murine Niemann-Pick C disease ameliorates neuronal cholesterol and glycosphingolipid storage and disease progression. PLoS ONE 4:e6951. 10.1371/journal.pone.0006951 19750228 PMC2736622

[B46] De Ru M. H. Boelens J. J. Das A. M. Jones S. A. van Der Lee J. H. Mahlaoui N.. (2011). Enzyme replacement therapy and/or hematopoietic stem cell transplantation at diagnosis in patients with mucopolysaccharidosis type I: results of a European consensus procedure. Orphanet J. Rare Dis. 6:55. 10.1186/1750-1172-6-55 21831279 PMC3170181

[B47] De Ruijter J. Valstar M. J. Narajczyk M. Wegrzyn G. Kulik W. Ijlst L.. (2012). Genistein in Sanfilippo disease: a randomized controlled crossover trial. Ann. Neurol. 71, 110–120. 10.1002/ana.22643 22275257

[B48] Del Grosso A. Galliani M. Angella L. Santi M. Tonazzini I. Parlanti G.. (2019). Brain-targeted enzyme-loaded nanoparticles: a breach through the blood-brain barrier for enzyme replacement therapy in Krabbe disease. Sci. Adv. 5:eaax7462. 10.1126/sciadv.aax7462 31799395 PMC6867879

[B49] Demeule M. Currie J. C. Bertrand Y. Ché C. Nguyen T. Régina A.. (2008). Involvement of the low-density lipoprotein receptor-related protein in the transcytosis of the brain delivery vector angiopep-2. J. Neurochem. 106, 1534–1544. 10.1111/j.1471-4159.2008.05492.x 18489712

[B50] Di Vizio D. Kim J. Hager M. H. Morello M. Yang W. Lafargue C. J.. (2009). Oncosome formation in prostate cancer: association with a region of frequent chromosomal deletion in metastatic disease. Cancer Res. 69, 5601–5609. 10.1158/0008-5472.CAN-08-3860 19549916 PMC2853876

[B51] Do M. A. Levy D. Brown A. Marriott G. Lu B. (2019). Targeted delivery of lysosomal enzymes to the endocytic compartment in human cells using engineered extracellular vesicles. Sci. Rep. 9:17274. 10.1038/s41598-019-53844-5 31754156 PMC6872767

[B52] Duffner P. K. Caviness V. S. Jr. Erbe R. W. Patterson M. C. Schultz K. R. Wenger D. A.. (2009). The long-term outcomes of presymptomatic infants transplanted for Krabbe disease: report of the workshop held on July 11 and 12, 2008, Holiday Valley, New York. Genet. Med 11, 450–454. 10.1097/GIM.0b013e3181a16e04 19346954

[B53] Durand S. Cougot N. Mahuteau-Betzer F. Nguyen C. H. Grierson D. S. Bertrand E.. (2007). Inhibition of nonsense-mediated mRNA decay (NMD) by a new chemical molecule reveals the dynamic of NMD factors in P-bodies. J. Cell Biol. 178, 1145–1160. 10.1083/jcb.200611086 17893241 PMC2064650

[B54] Eisengart J. B. Pierpont E. I. Kaizer A. M. Rudser K. D. King K. E. Pasquali M.. (2019). Intrathecal enzyme replacement for Hurler syndrome: biomarker association with neurocognitive outcomes. Genet. Med. 21, 2552–2560. 10.1038/s41436-019-0522-1 31019279 PMC6831510

[B55] Escolar M. L. Poe M. D. Provenzale J. M. Richards K. C. Allison J. Wood S.. (2005). Transplantation of umbilical-cord blood in babies with infantile Krabbe's disease. N. Engl. J. Med. 352, 2069–2081. 10.1056/NEJMoa042604 15901860

[B56] Fan Y. H. Yu Y. Mao R. F. Tan X. J. Xu G. F. Zhang H.. (2011). USP4 targets TAK1 to downregulate TNFα-induced NF-κB activation. Cell Death Differ. 18, 1547–1560. 10.1038/cdd.2011.11 21331078 PMC3136563

[B57] Farmer C. A. Thurm A. Farhat N. Bianconi S. Keener L. A. Porter F. D. (2019). Long-term neuropsychological outcomes from an open-label phase I/IIa trial of 2-hydroxypropyl-beta-cyclodextrins (VTS-270) in niemann-pick disease, type C1. CNS Drugs 33, 677–683. 10.1007/s40263-019-00642-2 31187454 PMC7448545

[B58] Foust K. D. Nurre E. Montgomery C. L. Hernandez A. Chan C. M. Kaspar B. K. (2009). Intravascular AAV9 preferentially targets neonatal neurons and adult astrocytes. Nat. Biotechnol. 27, 59–65. 10.1038/nbt.1515 19098898 PMC2895694

[B59] Fraldi A. Hemsley K. Crawley A. Lombardi A. Lau A. Sutherland L.. (2007). Functional correction of CNS lesions in an MPS-IIIA mouse model by intracerebral AAV-mediated delivery of sulfamidase and SUMF1 genes. Hum. Mol. Genet. 16, 2693–2702. 10.1093/hmg/ddm223 17725987

[B60] Fratantoni J. C. Hall C. W. Neufeld E. F. (1968). Hurler and hunter syndromes: mutual correction of the defect in cultured fibroblasts. Science 162, 570–572. 10.1126/science.162.3853.570 4236721

[B61] Frühbeis C. Fröhlich D. Krämer-Albers E. M. (2012). Emerging roles of exosomes in neuron-glia communication. Front. Physiol. 3:119. 10.3389/fphys.2012.00119 22557979 PMC3339323

[B62] Garnacho C. Dhami R. Simone E. Dziubla T. Leferovich J. Schuchman E. H.. (2008). Delivery of acid sphingomyelinase in normal and niemann-pick disease mice using intercellular adhesion molecule-1-targeted polymer nanocarriers. J. Pharmacol. Exp. Ther. 325, 400–408. 10.1124/jpet.107.133298 18287213

[B63] Gatto F. Rossi B. Tarallo A. Polishchuk E. Polishchuk R. Carrella A.. (2017). AAV-mediated transcription factor EB (TFEB) gene delivery ameliorates muscle pathology and function in the murine model of Pompe disease. Sci. Rep 7:15089. 10.1038/s41598-017-15352-2 29118420 PMC5678083

[B64] GBD 2016 Neurology Collaborators Group (2017). Global, regional, and national burden of neurological disorders during 1990-2015: a systematic analysis for the global burden of disease study 2015. Lancet Neurol. 16, 877–897. 10.1016/S1474-4422(17)30299-5 28931491 PMC5641502

[B65] Germain D. P. Hughes D. A. Nicholls K. Bichet D. G. Giugliani R. Wilcox W. R.. (2016). Treatment of Fabry's disease with the pharmacologic chaperone migalastat. N. Engl. J. Med. 375, 545–555. 10.1056/NEJMoa1510198 27509102

[B66] Ghose A. K. Herbertz T. Hudkins R. L. Dorsey B. D. Mallamo J. P. (2012). Knowledge-based, central nervous system (CNS) lead selection and lead optimization for CNS drug discovery. ACS Chem. Neurosci. 3, 50–68. 10.1021/cn200100h 22267984 PMC3260741

[B67] Ghosh P. Dahms N. M. Kornfeld S. (2003). Mannose 6-phosphate receptors: new twists in the tale. Nat. Rev. Mol. Cell Biol. 4, 202–212. 10.1038/nrm1050 12612639

[B68] Giannotti M. I. Esteban O. Oliva M. García-Parajo M. F. Sanz F. (2011). pH-responsive polysaccharide-based polyelectrolyte complexes as nanocarriers for lysosomal delivery of therapeutic proteins. Biomacromolecules 12, 2524–2533. 10.1021/bm2003384 21604696

[B69] Giraldo P. Alfonso P. Atutxa K. Fernandez-Galan M. A. Barez A. Franco R.. (2009). Real-world clinical experience with long-term miglustat maintenance therapy in type 1 Gaucher disease: the ZAGAL project. Haematologica 94, 1771–1775. 10.3324/haematol.2009.008078 19608672 PMC2791929

[B70] Giugliani R. Vairo F. Kubaski F. Poswar F. Riegel M. Baldo G.. (2018). Neurological manifestations of lysosomal disorders and emerging therapies targeting the CNS. Lancet Child Adolesc. Health 2, 56–68. 10.1016/S2352-4642(17)30087-1 30169196

[B71] Givogri M. I. Galbiati F. Fasano S. Amadio S. Perani L. Superchi D.. (2006). Oligodendroglial progenitor cell therapy limits central neurological deficits in mice with metachromatic leukodystrophy. J. Neurosci. 26, 3109–3119. 10.1523/JNEUROSCI.4366-05.2006 16554462 PMC6674100

[B72] Gleitz H. F. Liao A. Y. Cook J. R. Rowlston S. F. Forte G. M. D'souza Z.. (2018). Brain-targeted stem cell gene therapy corrects mucopolysaccharidosis type II via multiple mechanisms. EMBO Mol. Med. 10:e8730. 10.15252/emmm.201708730 29884617 PMC6034129

[B73] Graf W. D. (2017). Stem cell transplantation in Krabbe disease: new truths discovered and opinions change. Neurology 89, 1318–1319. 10.1212/WNL.0000000000004427 28855407

[B74] Gregoriadis G. (1978). Liposomes in the therapy of lysosomal storage diseases. Nature 275, 695–696. 10.1038/275695a0 100708

[B75] Gregoriadis G. Putman D. Louis L. Neerunjun D. (1974). Comparative effect and fate of non-entrapped and liposome-entrapped neuraminidase injected into rats. Biochem. J. 140, 323–330. 10.1042/bj1400323 4375965 PMC1168003

[B76] Groeschel S. Kuhl J. S. Bley A. E. Kehrer C. Weschke B. Doring M.. (2016). Long-term outcome of allogeneic hematopoietic stem cell transplantation in patients with juvenile metachromatic leukodystrophy compared with nontransplanted control patients. JAMA Neurol. 73, 1133–1140. 10.1001/jamaneurol.2016.2067 27400410

[B77] Guffon N. Bertrand Y. Forest I. Fouilhoux A. Froissart R. (2009). Bone marrow transplantation in children with Hunter syndrome: outcome after 7 to 17 years. J. Pediatr. 154, 733–737. 10.1016/j.jpeds.2008.11.041 19167723

[B78] Hamill K. M. Wexselblatt E. Tong W. Esko J. D. Tor Y. (2017). Delivery of cargo to lysosomes using GNeosomes. Methods Mol. Biol. 1594, 151–163. 10.1007/978-1-4939-6934-0_9 28456981

[B79] Han S. O. Li S. Everitt J. I. Koeberl D. D. (2019). Salmeterol with liver depot gene therapy enhances the skeletal muscle response in murine pompe disease. Hum. Gene Ther. 30, 855–864. 10.1089/hum.2018.197 30803275 PMC6648189

[B80] Haney M. J. Klyachko N. L. Harrison E. B. Zhao Y. Kabanov A. V. Batrakova E. V. (2019). TPP1 delivery to lysosomes with extracellular vesicles and their enhanced brain distribution in the animal model of batten disease. Adv. Healthc. Mater. 8:e1801271. 10.1002/adhm.201801271 30997751 PMC6584948

[B81] Hean J. Mager I. Wood M. El Andaloussi S. Wiklander O. Nordin J. (2019). Exosomes Comprising Therapeutic Polypeptides. Oxford: Google Patents.

[B82] Hinderer C. Bell P. Louboutin J. P. Zhu Y. Yu H. Lin G. (2015). Neonatal systemic AAV induces tolerance to CNS gene therapy in MPS I dogs and nonhuman primates. Mol. Ther. 23, 1298–1307. 10.1038/mt.2015.99 26022732 PMC4817868

[B83] Hocquemiller M. Giersch L. Audrain M. Parker S. Cartier N. (2016). Adeno-associated virus-based gene therapy for CNS diseases. Hum. Gene Ther. 27, 478–496. 10.1089/hum.2016.087 27267688 PMC4960479

[B84] Hollak C. E. Wijburg F. A. (2014). Treatment of lysosomal storage disorders: successes and challenges. J. Inherit. Metab. Dis. 37, 587–598. 10.1007/s10545-014-9718-3 24820227

[B85] Hsu J. Bhowmick T. Burks S. R. Kao J. P. Muro S. (2014). Enhancing biodistribution of therapeutic enzymes *in vivo* by modulating surface coating and concentration of ICAM-1-targeted nanocarriers. J. Biomed. Nanotechnol. 10, 345–354. 10.1166/jbn.2014.1718 24738342 PMC4000549

[B86] Hsu J. Northrup L. Bhowmick T. Muro S. (2012). Enhanced delivery of α-glucosidase for Pompe disease by ICAM-1-targeted nanocarriers: comparative performance of a strategy for three distinct lysosomal storage disorders. Nanomedicine 8, 731–739. 10.1016/j.nano.2011.08.014 21906578 PMC3279604

[B87] Hsu J. Serrano D. Bhowmick T. Kumar K. Shen Y. Kuo Y. C.. (2011). Enhanced endothelial delivery and biochemical effects of α-galactosidase by ICAM-1-targeted nanocarriers for Fabry disease. J. Control. Release 149, 323–331. 10.1016/j.jconrel.2010.10.031 21047542 PMC3073729

[B88] Hughes D. A. Nicholls K. Shankar S. P. Sunder-Plassmann G. Koeller D. Nedd K.. (2017). Oral pharmacological chaperone migalastat compared with enzyme replacement therapy in Fabry disease: 18-month results from the randomised phase III ATTRACT study. J. Med. Genet 54, 288–296. 10.1136/jmedgenet-2016-104178 27834756 PMC5502308

[B89] Huwyler J. Wu D. Pardridge W. M. (1996). Brain drug delivery of small molecules using immunoliposomes. Proc. Natl. Acad. Sci. U.S.A. 93, 14164–14169. 10.1073/pnas.93.24.14164 8943078 PMC19511

[B90] Jian J. Tian Q. Y. Hettinghouse A. Zhao S. Liu H. Wei J.. (2016). Progranulin recruits HSP70 to beta-glucocerebrosidase and is therapeutic against gaucher disease. EBioMedicine 13, 212–224. 10.1016/j.ebiom.2016.10.010 27789271 PMC5264254

[B91] Katabuchi A. U. Godoy V. Shil P. Moser A. Maegawa G. H. B. (2018). Serendipitous effects of beta-cyclodextrin on murine model of Krabbe disease. Mol Genet Metab Rep. 15, 98–99. 10.1016/j.ymgmr.2018.03.002 30023296 PMC6047113

[B92] Keeler G. D. Markusic D. M. Hoffman B. E. (2019). Liver induced transgene tolerance with AAV vectors. Cell. Immunol. 342:103728. 10.1016/j.cellimm.2017.12.002 29576315 PMC5988960

[B93] Kelly J. W. (2020). Pharmacologic approaches for adapting proteostasis in the secretory pathway to ameliorate protein conformational diseases. Cold Spring Harb. Perspect. Biol. 12, 1–19. 10.1101/cshperspect.a034108 31088828 PMC7197434

[B94] Kim G. Kim M. Lee Y. Byun J. W. Hwang D. W. Lee M. (2020). Systemic delivery of microRNA-21 antisense oligonucleotides to the brain using T7-peptide decorated exosomes. J. Control. Release 317, 273–281. 10.1016/j.jconrel.2019.11.009 31730913

[B95] Klionsky D. J. Abdelmohsen K. Abe A. Abedin M. J. Abeliovich H. Acevedo Arozena A.. (2016). Guidelines for the use and interpretation of assays for monitoring autophagy (3rd edition). Autophagy 12, 1–222. 10.1080/15548627.2015.1100356 26799652 PMC4835977

[B96] Koeberl D. D. Luo X. Sun B. Mcvie-Wylie A. Dai J. Li S.. (2011). Enhanced efficacy of enzyme replacement therapy in Pompe disease through mannose-6-phosphate receptor expression in skeletal muscle. Mol. Genet. Metab. 103, 107–112. 10.1016/j.ymgme.2011.02.006 21397538 PMC3101281

[B97] Koshkaryev A. Thekkedath R. Pagano C. Meerovich I. Torchilin V. P. (2011). Targeting of lysosomes by liposomes modified with octadecyl-rhodamine B. J. Drug Target 19, 606–614. 10.3109/1061186X.2010.550921 21275828 PMC4116792

[B98] Krageloh-Mann I. Groeschel S. Kehrer C. Opherk K. Nagele T. Handgretinger R.. (2013). Juvenile metachromatic leukodystrophy 10 years post transplant compared with a non-transplanted cohort. Bone Marrow Transplant. 48, 369–375. 10.1038/bmt.2012.155 22941383

[B99] Kwon J. M. Matern D. Kurtzberg J. Wrabetz L. Gelb M. H. Wenger D. A.. (2018). Consensus guidelines for newborn screening, diagnosis and treatment of infantile Krabbe disease. Orphanet J. Rare Dis 13:30. 10.1186/s13023-018-0766-x 29391017 PMC5796396

[B100] Lattanzi A. Neri M. Maderna C. Di Girolamo I. Martino S. Orlacchio A.. (2010). Widespread enzymatic correction of CNS tissues by a single intracerebral injection of therapeutic lentiviral vector in leukodystrophy mouse models. Hum. Mol. Genet. 19, 2208–2227. 10.1093/hmg/ddq099 20203170

[B101] Lebl R. Thonhofer M. Tysoe C. Pabst B. M. Schalli M. Weber P.. (2017). A Morita-Baylis-Hillman based route to C-5a-chain-extended 4-epi-isofagomine type glycosidase inhibitors. Carbohydr. Res. 442, 31–40. 10.1016/j.carres.2017.03.003 28288345

[B102] Lee H. J. Park H. H. Sohn Y. Ryu J. Park J. H. Rhee W. J.. (2016). α-Galactosidase delivery using 30Kc19-human serum albumin nanoparticles for effective treatment of Fabry disease. Appl. Microbiol. Biotechnol. 100, 10395–10402. 10.1007/s00253-016-7689-z 27353764

[B103] Low L. A. Sutherland M. Lumelsky N. Selimovic S. Lundberg M. S. Tagle D. A. (2020). Organs-on-a-chip. Adv. Exp. Med. Biol. 1230, 27–42. 10.1007/978-3-030-36588-2_3 32285363

[B104] Luddi A. Crifasi L. Capaldo A. Piomboni P. Costantino-Ceccarini E. (2016). Suppression of galactocerebrosidase premature termination codon and rescue of galactocerebrosidase activity in twitcher cells. J. Neurosci. Res. 94, 1273–1283. 10.1002/jnr.23790 27638609

[B105] Lund T. C. (2018). Umbilical cord blood expansion: are we there yet? Biol. Blood Marrow Transplant. 24, 1311–1312. 10.1016/j.bbmt.2018.05.002 29753835

[B106] Macias-Vidal J. Giros M. Guerrero M. Gascon P. Serratosa J. Bachs O.. (2014). The proteasome inhibitor bortezomib reduced cholesterol accumulation in fibroblasts from Niemann-Pick type C patients carrying missense mutations. FEBS J. 281, 4450–4466. 10.1111/febs.12954 25131710

[B107] Maegawa G. H. Banwell B. L. Blaser S. Sorge G. Toplak M. Ackerley C.. (2009a). Substrate reduction therapy in juvenile GM2 gangliosidosis. Mol. Genet. Metab. 98, 215–224. 10.1016/j.ymgme.2009.06.005 19595619

[B108] Maegawa G. H. Tropak M. Buttner J. Stockley T. Kok F. Clarke J. T.. (2007). Pyrimethamine as a potential pharmacological chaperone for late-onset forms of GM2 gangliosidosis. J. Biol. Chem. 282, 9150–9161. 10.1074/jbc.M609304200 17237499 PMC1851921

[B109] Maegawa G. H. Tropak M. B. Buttner J. D. Rigat B. A. Fuller M. Pandit D.. (2009b). Identification and characterization of ambroxol as an enzyme enhancement agent for Gaucher disease. J. Biol. Chem. 284, 23502–23516. 10.1074/jbc.M109.012393 19578116 PMC2749124

[B110] Maegawa G. H. Van Giersbergen P. L. Yang S. Banwell B. Morgan C. P. Dingemanse J.. (2009c). Pharmacokinetics, safety and tolerability of miglustat in the treatment of pediatric patients with GM2 gangliosidosis. Mol. Genet. Metab. 97, 284–291. 10.1016/j.ymgme.2009.04.013 19447653

[B111] Maegawa G. H. B. (2019). Lysosomal leukodystrophies lysosomal storage diseases associated with white matter abnormalities. J. Child Neurol. 34, 339–358. 10.1177/0883073819828587 30757954 PMC6459700

[B112] Magrin E. Miccio A. Cavazzana M. (2019). Lentiviral and genome-editing strategies for the treatment of beta-hemoglobinopathies. Blood 134, 1203–1213. 10.1182/blood.2019000949 31467062

[B113] Malinowska M. Wilkinson F. L. Bennett W. Langford-Smith K. J. O'leary H. A. Jakobkiewicz-Banecka J.. (2009). Genistein reduces lysosomal storage in peripheral tissues of mucopolysaccharide IIIB mice. Mol. Genet. Metab. 98, 235–242. 10.1016/j.ymgme.2009.06.013 19632871

[B114] Marco S. Haurigot V. Bosch F. (2019). *In vivo* gene therapy for mucopolysaccharidosis type III (Sanfilippo Syndrome): a new treatment horizon. Hum. Gene Ther. 30, 1211–1221. 10.1089/hum.2019.217 31482754

[B115] Markham A. (2017). Cerliponase alfa: first global approval. Drugs 77, 1247–1249. 10.1007/s40265-017-0771-8 28589525

[B116] Marshall J. Sun Y. Bangari D. S. Budman E. Park H. Nietupski J. B.. (2016). CNS-accessible inhibitor of glucosylceramide synthase for substrate reduction therapy of neuronopathic gaucher disease. Mol. Ther. 24, 1019–1029. 10.1038/mt.2016.53 26948439 PMC4923322

[B117] Matalonga L. Gort L. Ribes A. (2017). Small molecules as therapeutic agents for inborn errors of metabolism. J. Inherit. Metab. Dis. 40, 177–193. 10.1007/s10545-016-0005-3 27966099

[B118] Mayer F. Q. Artigalas O. A. Lagranha V. L. Baldo G. Schwartz I. V. Matte U.. (2013). Chloramphenicol enhances IDUA activity on fibroblasts from mucopolysaccharidosis I patients. Curr. Pharm. Biotechnol. 14, 194–198. 10.2174/1389201011314020009 23167761

[B119] Mceachern K. A. Fung J. Komarnitsky S. Siegel C. S. Chuang W. L. Hutto E.. (2007). A specific and potent inhibitor of glucosylceramide synthase for substrate inhibition therapy of Gaucher disease. Mol. Genet. Metab. 91, 259–267. 10.1016/j.ymgme.2007.04.001 17509920

[B120] Mcgraw P. Liang L. Escolar M. Mukundan S. Kurtzberg J. Provenzale J. M. (2005). Krabbe disease treated with hematopoietic stem cell transplantation: serial assessment of anisotropy measurements–initial experience. Radiology 236, 221–230. 10.1148/radiol.2353040716 15987975

[B121] Mckinnis E. J. Sulzbacher S. Rutledge J. C. Sanders J. Scott C. R. (1996). Bone marrow transplantation in Hunter syndrome. J. Pediatr. 129, 145–148. 10.1016/S0022-3476(96)70202-0 8757575

[B122] Meneghini V. Lattanzi A. Tiradani L. Bravo G. Morena F. Sanvito F.. (2016). Pervasive supply of therapeutic lysosomal enzymes in the CNS of normal and Krabbe-affected non-human primates by intracerebral lentiviral gene therapy. EMBO Mol. Med. 8, 489–510. 10.15252/emmm.201505850 27025653 PMC5128736

[B123] Meng X. Xu G. Zhou Q.-L. Wu J.-P. Yang L.-R. (2013). Improvements of lipase performance in high-viscosity system by immobilization onto a novel kind of poly (methylmethacrylate-co-divinylbenzene) encapsulated porous magnetic microsphere carrier. J. Mol. Catal. B Enzymatic 89, 86–92. 10.1016/j.molcatb.2013.01.006

[B124] Minciacchi V. R. You S. Spinelli C. Morley S. Zandian M. Aspuria P. J.. (2015). Large oncosomes contain distinct protein cargo and represent a separate functional class of tumor-derived extracellular vesicles. Oncotarget 6, 11327–11341. 10.18632/oncotarget.3598 25857301 PMC4484459

[B125] Mistry P. K. Lukina E. Ben Turkia H. Amato D. Baris H. Dasouki M.. (2015). Effect of oral eliglustat on splenomegaly in patients with Gaucher disease type 1: the ENGAGE randomized clinical trial. JAMA 313, 695–706. 10.1001/jama.2015.459 25688781 PMC4962880

[B126] Mort M. Ivanov D. Cooper D. N. Chuzhanova N. A. (2008). A meta-analysis of nonsense mutations causing human genetic disease. Hum. Mutat. 29, 1037–1047. 10.1002/humu.20763 18454449

[B127] Mu T. W. Fowler D. M. Kelly J. W. (2008a). Partial restoration of mutant enzyme homeostasis in three distinct lysosomal storage disease cell lines by altering calcium homeostasis. PLoS Biol. 6:e26. 10.1371/journal.pbio.0060026 18254660 PMC2225441

[B128] Mu T. W. Ong D. S. Wang Y. J. Balch W. E. Yates J. R. 3rd, Segatori, L. Kelly J. W. (2008b). Chemical and biological approaches synergize to ameliorate protein-folding diseases. Cell 134, 769–781. 10.1016/j.cell.2008.06.037 18775310 PMC2650088

[B129] Muenzer J. Beck M. Eng C. M. Escolar M. L. Giugliani R. Guffon N. H.. (2009a). Multidisciplinary management of Hunter syndrome. Pediatrics 124, e1228–e1239. 10.1542/peds.2008-0999 19901005

[B130] Muenzer J. Hendriksz C. J. Fan Z. Vijayaraghavan S. Perry V. Santra S.. (2016). A phase I/II study of intrathecal idursulfase-IT in children with severe mucopolysaccharidosis II. Genet. Med. 18, 73–81. 10.1038/gim.2015.36 25834948

[B131] Muenzer J. Wraith J. E. Clarke L. A. International Consensus Panel On M. Treatment of Mucopolysaccharidosis I. (2009b). Mucopolysaccharidosis I: management and treatment guidelines. Pediatrics 123, 19–29. 10.1542/peds.2008-0416 19117856

[B132] Munoz-Rojas M. V. Vieira T. Costa R. Fagondes S. John A. Jardim L. B.. (2008). Intrathecal enzyme replacement therapy in a patient with mucopolysaccharidosis type I and symptomatic spinal cord compression. Am. J. Med. Genet. A 146A, 2538–2544. 10.1002/ajmg.a.32294 18792977

[B133] Muro S. Schuchman E. H. Muzykantov V. R. (2006). Lysosomal enzyme delivery by ICAM-1-targeted nanocarriers bypassing glycosylation- and clathrin-dependent endocytosis. Mol. Ther. 13, 135–141. 10.1016/j.ymthe.2005.07.687 16153895

[B134] Naldini L. (2019). Genetic engineering of hematopoiesis: current stage of clinical translation and future perspectives. EMBO Mol. Med. 11:e9985. 10.15252/emmm.201809958 30670463 PMC6404113

[B135] Narita A. Shirai K. Itamura S. Matsuda A. Ishihara A. Matsushita K.. (2016). Ambroxol chaperone therapy for neuronopathic Gaucher disease: a pilot study. Ann. Clin. Transl. Neurol. 3, 200–215. 10.1002/acn3.292 27042680 PMC4774255

[B136] Nestrasil I. Shapiro E. Svatkova A. Dickson P. Chen A. Wakumoto A.. (2017). Intrathecal enzyme replacement therapy reverses cognitive decline in mucopolysaccharidosis type I. Am. J. Med. Genet. A 173, 780–783. 10.1002/ajmg.a.38073 28211988 PMC5367919

[B137] Neufeld E. F. (1989). Natural history and inherited disorders of a lysosomal enzyme, beta-hexosaminidase. J. Biol. Chem 264, 10927–10930. 2525553

[B138] Noerholm M. Balaj L. Limperg T. Salehi A. Zhu L. D. Hochberg F. H.. (2012). RNA expression patterns in serum microvesicles from patients with glioblastoma multiforme and controls. BMC Cancer 12:22. 10.1186/1471-2407-12-22 22251860 PMC3329625

[B139] Nolte-'T Hoen E. N. van Der Vlist E. J. Aalberts M. Mertens H. C. Bosch B. J. Bartelink W.. (2012). Quantitative and qualitative flow cytometric analysis of nanosized cell-derived membrane vesicles. Nanomedicine 8, 712–720. 10.1016/j.nano.2011.09.006 22024193 PMC7106164

[B140] O'loughlin A. J. Woffindale C. A. Wood M. J. (2012). Exosomes and the emerging field of exosome-based gene therapy. Curr. Gene Ther. 12, 262–274. 10.2174/156652312802083594 22856601

[B141] Onaca-Fischer O. Liu J. Inglin M. Palivan C. G. (2012). Polymeric nanocarriers and nanoreactors: a survey of possible therapeutic applications. Curr. Pharm. Des. 18, 2622–2643. 10.2174/138161212800492822 22512447

[B142] Ory D. S. Ottinger E. A. Farhat N. Y. King K. A. Jiang X. Weissfeld L.. (2017). Intrathecal 2-hydroxypropyl-beta-cyclodextrin decreases neurological disease progression in Niemann-Pick disease, type C1: a non-randomised, open-label, phase 1-2 trial. Lancet 390, 1758–1768. 10.1016/S0140-6736(17)31465-4 28803710 PMC6176479

[B143] Osher E. Fattal-Valevski A. Sagie L. Urshanski N. Amir-Levi Y. Katzburg S.. (2011). Pyrimethamine increases beta-hexosaminidase A activity in patients with late onset tay Sachs. Mol. Genet. Metab. 102, 356–363. 10.1016/j.ymgme.2010.11.163 21185210

[B144] Ou L. Przybilla M. J. Koniar B. Whitley C. B. (2018). RTB lectin-mediated delivery of lysosomal alpha-l-iduronidase mitigates disease manifestations systemically including the central nervous system. Mol. Genet. Metab. 123, 105–111. 10.1016/j.ymgme.2017.11.013 29198892 PMC5808854

[B145] Palmieri M. Pal R. Nelvagal H. R. Lotfi P. Stinnett G. R. Seymour M. L. (2017). mTORC1-independent TFEB activation via Akt inhibition promotes cellular clearance in neurodegenerative storage diseases. Nat. Commun. 8:14338 10.1038/ncomms15793 28165011 PMC5303831

[B146] Pardridge W. M. (2001). Crossing the blood-brain barrier: are we getting it right? Drug Discov. Today 6, 1–2. 10.1016/S1359-6446(00)01583-X 11165157

[B147] Pardridge W. M. (2005). The blood-brain barrier and neurotherapeutics. NeuroRx 2, 1–2. 10.1602/neurorx.2.1.1 15717052 PMC539315

[B148] Pardridge W. M. (2006). Molecular Trojan horses for blood-brain barrier drug delivery. Curr. Opin. Pharmacol 6, 494–500. 10.1016/j.coph.2006.06.001 16839816

[B149] Pardridge W. M. (2007a). Blood-brain barrier delivery. Drug Discov. Today 12, 54–61. 10.1016/j.drudis.2006.10.013 17198973

[B150] Pardridge W. M. (2007b). Blood-brain barrier delivery of protein and non-viral gene therapeutics with molecular Trojan horses. J. Control. Release 122, 345–348. 10.1016/j.jconrel.2007.04.001 17512078 PMC2701689

[B151] Park H. H. Sohn Y. Yeo J. W. Park J. H. Lee H. J. Ryu J.. (2014). Dimerization of 30Kc19 protein in the presence of amphiphilic moiety and importance of Cys-57 during cell penetration. Biotechnol. J. 9, 1582–1593. 10.1002/biot.201400253 25143246 PMC4283735

[B152] Park J. H. Lee J. H. Park H. H. Rhee W. J. Choi S. S. Park T. H. (2012). A protein delivery system using 30Kc19 cell-penetrating protein originating from silkworm. Biomaterials 33, 9127–9134. 10.1016/j.biomaterials.2012.08.063 22981778

[B153] Pastores G. M. Elstein D. Hrebicek M. Zimran A. (2007). Effect of miglustat on bone disease in adults with type 1 Gaucher disease: a pooled analysis of three multinational, open-label studies. Clin. Ther. 29, 1645–1654. 10.1016/j.clinthera.2007.08.006 17919546

[B154] Pastores G. M. Maegawa G. H. (2013). Clinical neurogenetics: neuropathic lysosomal storage disorders. Neurol. Clin. 31, 1051–1071. 10.1016/j.ncl.2013.04.007 24176423 PMC3988112

[B155] Patel T. Zhou J. Piepmeier J. M. Saltzman W. M. (2012). Polymeric nanoparticles for drug delivery to the central nervous system. Adv. Drug Deliv. Rev. 64, 701–705. 10.1016/j.addr.2011.12.006 22210134 PMC3323692

[B156] Patel H. Ryman B. E. (1974). α-Mannosidase in Zinc-Deficient Rats: Possibility of Liposomal Therapy in Annosidosis. London: Portland Press Ltd 10.1042/bst0021014

[B157] Patil S. A. Maegawa G. H. (2013). Developing therapeutic approaches for metachromatic leukodystrophy. Drug Des. Devel. Ther. 7, 729–745. 10.2147/DDDT.S15467 23966770 PMC3743609

[B158] Patterson M. C. Vecchio D. Prady H. Abel L. Wraith J. E. (2007). Miglustat for treatment of Niemann-Pick C disease: a randomised controlled study. Lancet Neurol. 6, 765–772. 10.1016/S1474-4422(07)70194-1 17689147

[B159] Pawlinski L. Malecki M. T. Kiec-Wilk B. (2016). The additive effect on the antiepileptic treatment of ambroxol in type 3 Gaucher patient. The early observation. Blood Cells Mol. Dis. 11, 192–193. 10.1016/j.bcmd.2016.12.001 28007403

[B160] Peled T. Landau E. Mandel J. Glukhman E. Goudsmid N. R. Nagler A.. (2004). Linear polyamine copper chelator tetraethylenepentamine augments long-term *ex vivo* expansion of cord blood-derived CD34+ cells and increases their engraftment potential in NOD/SCID mice. Exp. Hematol. 32, 547–555. 10.1016/j.exphem.2004.03.002 15183895

[B161] Peng J. Dalton J. Butt M. Tracy K. Kennedy D. Haroldsen P.. (2017). Reveglucosidase alfa (BMN 701), an IGF2-tagged rhacid alpha-glucosidase, improves respiratory functional parameters in a murine model of pompe disease. J. Pharmacol. Exp. Ther. 360, 313–323. 10.1124/jpet.116.235952 27856936

[B162] Pipalia N. H. Subramanian K. Mao S. Ralph H. Hutt D. M. Scott S. M.. (2017). Histone deacetylase inhibitors correct the cholesterol storage defect in most Niemann-Pick C1 mutant cells. J. Lipid Res. 58, 695–708. 10.1194/jlr.M072140 28193631 PMC5392745

[B163] Pirooznia N. Hasannia S. Lotfi A. S. Ghanei M. (2012). Encapsulation of alpha-1 antitrypsin in PLGA nanoparticles: *in vitro* characterization as an effective aerosol formulation in pulmonary diseases. J. Nanobiotechnology 10:20. 10.1186/1477-3155-10-20 22607686 PMC3485170

[B164] Platt F. M. (2018). Emptying the stores: lysosomal diseases and therapeutic strategies. Nat. Rev. Drug Discov. 17, 133–150. 10.1038/nrd.2017.214 29147032

[B165] Platt F. M. D'azzo A. Davidson B. L. Neufeld E. F. Tifft C. J. (2018). Lysosomal storage diseases. Nat. Rev. Dis. Primers 4:27 10.1038/s41572-018-0037-0 30275469

[B166] Platt F. M. Jeyakumar M. (2008). Substrate reduction therapy. Acta Paediatr. Suppl. 97, 88–93. 10.1111/j.1651-2227.2008.00656.x 18339196

[B167] Platt F. M. Neises G. R. Dwek R. A. Butters T. D. (1994a). N-butyldeoxynojirimycin is a novel inhibitor of glycolipid biosynthesis. J. Biol. Chem. 269, 8362–8365. 8132559

[B168] Platt F. M. Neises G. R. Karlsson G. B. Dwek R. A. Butters T. D. (1994b). N-butyldeoxygalactonojirimycin inhibits glycolipid biosynthesis but does not affect N-linked oligosaccharide processing. J. Biol. Chem. 269, 27108–27114. 7929454

[B169] Platt F. M. Reinkensmeier G. Dwek R. A. Butters T. D. (1997). Extensive glycosphingolipid depletion in the liver and lymphoid organs of mice treated with N-butyldeoxynojirimycin. J. Biol. Chem. 272, 19365–19372. 10.1074/jbc.272.31.19365 9235935

[B170] Powers E. T. Balch W. E. (2013). Diversity in the origins of proteostasis networks–a driver for protein function in evolution. Nat. Rev. Mol. Cell Biol. 14, 237–248. 10.1038/nrm3542 23463216 PMC3718298

[B171] Prasad V. K. Kurtzberg J. (2008). Emerging trends in transplantation of inherited metabolic diseases. Bone Marrow Transplant. 41, 99–108. 10.1038/sj.bmt.1705970 18176609

[B172] Prasad V. K. Kurtzberg J. (2010a). Cord blood and bone marrow transplantation in inherited metabolic diseases: scientific basis, current status and future directions. Br. J. Haematol. 148, 356–372. 10.1111/j.1365-2141.2009.07974.x 19919654

[B173] Prasad V. K. Kurtzberg J. (2010b). Transplant outcomes in mucopolysaccharidoses. Semin. Hematol. 47, 59–69. 10.1053/j.seminhematol.2009.10.008 20109613

[B174] Prill H. Luu A. Yip B. Holtzinger J. Lo M. J. Christianson T. M.. (2019). Differential uptake of NAGLU-IGF2 and unmodified NAGLU in cellular models of sanfilippo syndrome Type, B. Mol. Ther. Methods Clin. Dev. 14, 56–63. 10.1016/j.omtm.2019.05.008 31309128 PMC6606967

[B175] Rajendran L. Honsho M. Zahn T. R. Keller P. Geiger K. D. Verkade P.. (2006). Alzheimer's disease beta-amyloid peptides are released in association with exosomes. Proc. Natl. Acad. Sci. U. S. A. 103, 11172–11177. 10.1073/pnas.0603838103 16837572 PMC1544060

[B176] Raposo G. Stoorvogel W. (2013). Extracellular vesicles: exosomes, microvesicles, and friends. J. Cell Biol. 200, 373–383. 10.1083/jcb.201211138 23420871 PMC3575529

[B177] Ricca A. Cascino F. Morena F. Martino S. Gritti A. (2020). *In vitro* validation of chimeric beta-Galactosylceramidase enzymes with improved enzymatic activity and increased secretion. Front Mol Biosci. 7:167. 10.3389/fmolb.2020.00167 32850960 PMC7396597

[B178] Roth D. M. Balch W. E. (2011). Modeling general proteostasis: proteome balance in health and disease. Curr. Opin. Cell Biol. 23, 126–134. 10.1016/j.ceb.2010.11.001 21131189 PMC3077458

[B179] Salvalaio M. Rigon L. Belletti D. D'avanzo F. Pederzoli F. Ruozi B.. (2016). Targeted polymeric nanoparticles for brain delivery of high molecular weight molecules in lysosomal storage disorders. PLoS ONE 11:e0156452. 10.1371/journal.pone.0156452 27228099 PMC4881964

[B180] Sandhoff K. Conzelmann E. (1984). The biochemical basis of gangliosidoses. Neuropediatrics 15 (Suppl.):85–92. 10.1055/s-2008-1052387 6242704

[B181] Santi M. Maccari G. Mereghetti P. Voliani V. Rocchiccioli S. Ucciferri N.. (2017). Rational design of a transferrin-binding peptide sequence tailored to targeted nanoparticle internalization. Bioconjug. Chem. 28, 471–480. 10.1021/acs.bioconjchem.6b00611 27977155

[B182] Sarrazin S. Wilson B. Sly W. S. Tor Y. Esko J. D. (2010). Guanidinylated neomycin mediates heparan sulfate-dependent transport of active enzymes to lysosomes. Mol. Ther. 18, 1268–1274. 10.1038/mt.2010.78 20442709 PMC2911259

[B183] Sawkar A. R. Schmitz M. Zimmer K. P. Reczek D. Edmunds T. Balch W. E.. (2006). Chemical chaperones and permissive temperatures alter localization of Gaucher disease associated glucocerebrosidase variants. ACS Chem. Biol. 1, 235–251. 10.1021/cb600187q 17163678

[B184] Scarpa M. Almassy Z. Beck M. Bodamer O. Bruce I. A. de Meirleir L.. (2011). Mucopolysaccharidosis type II: European recommendations for the diagnosis and multidisciplinary management of a rare disease. Orphanet J. Rare Dis. 6:72. 10.1186/1750-1172-6-72 22059643 PMC3223498

[B185] Schiffmann R. Fitzgibbon E. J. Harris C. Devile C. Davies E. H. Abel L.. (2008). Randomized, controlled trial of miglustat in Gaucher's disease type 3. Ann. Neurol. 64, 514–522. 10.1002/ana.21491 19067373 PMC2605167

[B186] Schueler U. H. Kolter T. Kaneski C. R. Zirzow G. C. Sandhoff K. Brady R. O. (2004). Correlation between enzyme activity and substrate storage in a cell culture model system for Gaucher disease. J. Inherit. Metab. Dis. 27, 649–658. 10.1023/B:BOLI.0000042959.44318.7c 15669681

[B187] Schulz A. Ajayi T. Specchio N. de Los Reyes E. Gissen P. Ballon D.. (2018). Study of intraventricular cerliponase Alfa for CLN2 disease. N. Engl. J. Med. 378, 1898–1907. 10.1056/NEJMoa1712649 29688815

[B188] Sessa M. Lorioli L. Fumagalli F. Acquati S. Redaelli D. Baldoli C.. (2016). Lentiviral haemopoietic stem-cell gene therapy in early-onset metachromatic leukodystrophy: an ad-hoc analysis of a non-randomised, open-label, phase 1/2 trial. Lancet 388, 476–487. 10.1016/S0140-6736(16)30374-9 27289174

[B189] Shapiro B. E. Pastores G. M. Gianutsos J. Luzy C. Kolodny E. H. (2009). Miglustat in late-onset Tay-Sachs disease: a 12-month, randomized, controlled clinical study with 24 months of extended treatment. Genet. Med. 11, 425–433. 10.1097/GIM.0b013e3181a1b5c5 19346952

[B190] Shayman J. A. (2010). Eliglustat tartrate: glucosylceramide synthase inhibitor treatment of type 1 gaucher disease. Drugs Future 35, 613–620. 10.1358/dof.2010.035.08.1505566 22563139 PMC3340614

[B191] Shayman J. A. (2013). The design and clinical development of inhibitors of glycosphingolipid synthesis: will invention be the mother of necessity? Trans. Am. Clin. Climatol. Assoc. 124, 46–60. 23874009 PMC3715929

[B192] Shihabuddin L. S. Numan S. Huff M. R. Dodge J. C. Clarke J. Macauley S. L.. (2004). Intracerebral transplantation of adult mouse neural progenitor cells into the Niemann-Pick-A mouse leads to a marked decrease in lysosomal storage pathology. J. Neurosci. 24, 10642–10651. 10.1523/JNEUROSCI.3584-04.2004 15564580 PMC6730128

[B193] Song W. Wang F. Savini M. Ake A. Di Ronza A. Sardiello M.. (2013). TFEB regulates lysosomal proteostasis. Hum. Mol. Genet 22, 1994–2009. 10.1093/hmg/ddt052 23393155

[B194] Sorrentino N. C. D'orsi L. Sambri I. Nusco E. Monaco C. Spampanato C.. (2013). A highly secreted sulphamidase engineered to cross the blood-brain barrier corrects brain lesions of mice with mucopolysaccharidoses type IIIA. EMBO Mol. Med. 5, 675–690. 10.1002/emmm.201202083 23568409 PMC3662312

[B195] Springer T. A. (1994). Traffic signals for lymphocyte recirculation and leukocyte emigration: the multistep paradigm. Cell 76, 301–314. 10.1016/0092-8674(94)90337-9 7507411

[B196] Steger L. D. Desnick R. J. (1977). Enzyme therapy. VI: comparative *in vivo* fates and effects on lysosomal integrity of enzyme entrapped in negatively and positively charged liposomes. Biochim. Biophys. Acta 464, 530–546. 10.1016/0005-2736(77)90028-1 836826

[B197] Stiff P. J. Montesinos P. Peled T. Landau E. Goudsmid N. R. Mandel J.. (2018). Cohort-controlled comparison of umbilical cord blood transplantation using carlecortemcel-L, a single progenitor-enriched cord blood, to double cord blood unit transplantation. Biol. Blood Marrow Transplant. 24, 1463–1470. 10.1016/j.bbmt.2018.02.012 29477778 PMC6045964

[B198] Sun Y. Liou B. Chu Z. Fannin V. Blackwood R. Peng Y.. (2020). Systemic enzyme delivery by blood-brain barrier-penetrating SapC-DOPS nanovesicles for treatment of neuronopathic Gaucher disease. EBioMedicine 2020:102735. 10.1016/j.ebiom.2020.102735 32279952 PMC7251241

[B199] Sun Y. Qi X. Grabowski G. A. (2003). Saposin C is required for normal resistance of acid beta-glucosidase to proteolytic degradation. J. Biol. Chem. 278, 31918–31923. 10.1074/jbc.M302752200 12813057

[B200] Sweeney M. D. Sagare A. P. Zlokovic B. V. (2018). Blood-brain barrier breakdown in Alzheimer disease and other neurodegenerative disorders. Nat. Rev. Neurol. 14, 133–150. 10.1038/nrneurol.2017.188 29377008 PMC5829048

[B201] Tamaki S. J. Jacobs Y. Dohse M. Capela A. Cooper J. D. Reitsma M.. (2009). Neuroprotection of host cells by human central nervous system stem cells in a mouse model of infantile neuronal ceroid lipofuscinosis. Cell Stem Cell 5, 310–319. 10.1016/j.stem.2009.05.022 19733542

[B202] Tan E. Y. Boelens J. J. Jones S. A. Wynn R. F. (2019). Hematopoietic stem cell transplantation in inborn errors of metabolism. Front. Pediatr. 7:433. 10.3389/fped.2019.00433 31709204 PMC6824291

[B203] Tardieu M. Zerah M. Husson B. de Bournonville S. Deiva K. Adamsbaum C.. (2014). Intracerebral administration of adeno-associated viral vector serotype rh.10 carrying human SGSH and SUMF1 cDNAs in children with mucopolysaccharidosis type IIIA disease: results of a phase I/II trial. Hum. Gene Ther. 25, 506–516. 10.1089/hum.2013.238 24524415

[B204] Tejera E. Rocha-Perugini V. López-Martín S. Pérez-Hernández D. Bachir A. I. Horwitz A. R.. (2013). CD81 regulates cell migration through its association with Rac GTPase. Mol. Biol. Cell 24, 261–273. 10.1091/mbc.e12-09-0642 23264468 PMC3564539

[B205] Thada V. Miller J. N. Kovacs A. D. Pearce D. A. (2016). Tissue-specific variation in nonsense mutant transcript level and drug-induced read-through efficiency in the Cln1(R151X) mouse model of INCL. J. Cell. Mol. Med. 20, 381–385. 10.1111/jcmm.12744 26648046 PMC4727554

[B206] Thekkedath R. Koshkaryev A. Torchilin V. P. (2013). Lysosome-targeted octadecyl-rhodamine B-liposomes enhance lysosomal accumulation of glucocerebrosidase in Gaucher's cells *in vitro*. Nanomedicine 8, 1055–1065. 10.2217/nnm.12.138 23199221 PMC3644353

[B207] Théry C. Zitvogel L. Amigorena S. (2002). Exosomes: composition, biogenesis and function. Nat. Rev. Immunol. 2, 569–579. 10.1038/nri855 12154376

[B208] Torra A. Parent A. Cuadros T. Rodriguez-Galvan B. Ruiz-Bronchal E. Ballabio A.. (2018). Overexpression of TFEB drives a pleiotropic neurotrophic effect and prevents parkinson's disease-related neurodegeneration. Mol. Ther. 26, 1552–1567. 10.1016/j.ymthe.2018.02.022 29628303 PMC5986717

[B209] Tosi G. Fano R. A. Bondioli L. Badiali L. Benassi R. Rivasi F. (2011). Investigation on mechanisms of glycopeptide nanoparticles for drug delivery across the blood-brain barrier. Nanomedicine 6, 423–436. 10.2217/nnm.11.11 21542682

[B210] Tropak M. B. Mahuran D. (2007). Lending a helping hand, screening chemical libraries for compounds that enhance beta-hexosaminidase A activity in GM2 gangliosidosis cells. FEBS J. 274, 4951–4961. 10.1111/j.1742-4658.2007.06040.x 17894780 PMC2910757

[B211] Umezawa F. Eto Y. Tokoro T. Ito F. Maekawa K. (1985). Enzyme replacement with liposomes containing beta-galactosidase from Charonia lumpas in murine globoid cell leukodystrophy (twitcher). Biochem. Biophys. Res. Commun. 127, 663–667. 10.1016/S0006-291X(85)80212-6 3919736

[B212] Van Niel G. D'angelo G. Raposo G. (2018). Shedding light on the cell biology of extracellular vesicles. Nat. Rev. Mol. Cell Biol. 19, 213–228. 10.1038/nrm.2017.125 29339798

[B213] Van Rappard D. F. Boelens J. J. van Egmond M. E. Kuball J. Van Hasselt P. M. Oostrom K. J.. (2016). Efficacy of hematopoietic cell transplantation in metachromatic leukodystrophy: the Dutch experience. Blood 127, 3098–3101. 10.1182/blood-2016-03-708479 27118454

[B214] Walkley S. U. (2009). Pathogenic cascades in lysosomal disease-why so complex? J. Inherit. Metab. Dis. 32, 181–189. 10.1007/s10545-008-1040-5 19130290 PMC2682782

[B215] Wang D. Belakhov V. Kandasamy J. Baasov T. Li S. C. Li Y. T.. (2012). The designer aminoglycoside NB84 significantly reduces glycosaminoglycan accumulation associated with MPS I-H in the Idua-W392X mouse. Mol. Genet. Metab. 105, 116–125. 10.1016/j.ymgme.2011.10.005 22056610 PMC3253910

[B216] Wang D. El-Amouri S. S. Dai M. Kuan C. Y. Hui D. Y. Brady R. O.. (2013). Engineering a lysosomal enzyme with a derivative of receptor-binding domain of apoE enables delivery across the blood-brain barrier. Proc. Natl. Acad. Sci. U. S. A. 110, 2999–3004. 10.1073/pnas.1222742110 23382178 PMC3581871

[B217] Wang F. Segatori L. (2013). Remodeling the proteostasis network to rescue glucocerebrosidase variants by inhibiting ER-associated degradation and enhancing ER Folding. PLoS ONE 8:e61418. 10.1371/journal.pone.0061418 23620750 PMC3631227

[B218] Wasserstein M. P. Diaz G. A. Lachmann R. H. Jouvin M. H. Nandy I. Ji A. J.. (2018). Olipudase alfa for treatment of acid sphingomyelinase deficiency (ASMD): safety and efficacy in adults treated for 30 months. J. Inherit. Metab. Dis. 41, 829–838. 10.1007/s10545-017-0123-6 29305734 PMC6133173

[B219] Whitley C. B. Vijay S. Yao B. Pineda M. Parker G. J. M. Rojas-Caro S.. (2019). Final results of the phase 1/2, open-label clinical study of intravenous recombinant human N-acetyl-alpha-d-glucosaminidase (SBC-103) in children with mucopolysaccharidosis IIIB. Mol. Genet. Metab. 126, 131–138. 10.1016/j.ymgme.2018.12.003 30635159

[B220] Wiseman D. H. Mercer J. Tylee K. Malaiya N. Bonney D. K. Jones S. A.. (2013). Management of mucopolysaccharidosis type IH (Hurler's syndrome) presenting in infancy with severe dilated cardiomyopathy: a single institution's experience. J. Inherit. Metab. Dis. 36, 263–270. 10.1007/s10545-012-9500-3 22718273

[B221] Wiseman R. L. Powers E. T. Buxbaum J. N. Kelly J. W. Balch W. E. (2007). An adaptable standard for protein export from the endoplasmic reticulum. Cell 131, 809–821. 10.1016/j.cell.2007.10.025 18022373

[B222] Xu S. Lun Y. Frascella M. Garcia A. Soska R. Nair A.. (2019). Improved efficacy of a next-generation ERT in murine Pompe disease. JCI Insight. 4:e125358. 10.1172/jci.insight.125358 30843882 PMC6483515

[B223] Yang J. Zhang X. Chen X. Wang L. Yang G. (2017). Exosome mediated delivery of miR-124 promotes neurogenesis after Ischemia. Mol. Ther. Nucleic Acids 7, 278–287. 10.1016/j.omtn.2017.04.010 28624203 PMC5415550

[B224] Zhang G. Yang P. (2018). A novel cell-cell communication mechanism in the nervous system: exosomes. J. Neurosci. Res. 96, 45–52. 10.1002/jnr.24113 28718905

[B225] Zipkin M. (2019). Exosome redux. Nat. Biotechnol. 37, 1395–1400 10.1038/s41587-019-0326-5 31796920

